# Radiotherapy‐Induced Astrocyte Senescence Promotes an Immunosuppressive Microenvironment in Glioblastoma to Facilitate Tumor Regrowth

**DOI:** 10.1002/advs.202304609

**Published:** 2024-02-11

**Authors:** Jianxiong Ji, Kaikai Ding, Bo Cheng, Xin Zhang, Tao Luo, Bin Huang, Hao Yu, Yike Chen, Xiaohui Xu, Haopu Lin, Jiayin Zhou, Tingtin Wang, Mengmeng Jin, Aixia Liu, Danfang Yan, Fuyi Liu, Chun Wang, Jingsen Chen, Feng Yan, Lin Wang, Jianmin Zhang, Senxiang Yan, Jian Wang, Xingang Li, Gao Chen

**Affiliations:** ^1^ Department of Neurosurgery the Second Affiliated Hospital of Zhejiang University School of Medicine Key Laboratory of Precise Treatment and Clinical Translational Research of Neurological Diseases Hangzhou Zhejiang 310000 P. R. China; ^2^ Department of Neurosurgery Qilu Hospital of Shandong University and Brain Science Research Institute Cheeloo College of Medicine Shandong University 107 Wenhua Xi Road Jinan Shandong 250012 P. R. China; ^3^ Key Laboratory of Brain Functional Remodeling Shandong University 107 Wenhua Xi Road Jinan Shandong 250012 P. R. China; ^4^ Department of Radiation Oncology Mayo Clinic Rochester MN 55905 USA; ^5^ Department of Radiation Oncology the First Affiliated Hospital School of Medicine Zhejiang University Hangzhou Zhejiang 310000 P. R. China; ^6^ Department of Radiation Oncology Qilu Hospital of Shandong University Cheeloo College of Medicine Shandong University Jinan Shandong 250012 P. R. China; ^7^ Department of Reproductive Endocrinology Women's Hospital Zhejiang University School of Medicine Hangzhou Zhejiang 310000 P. R. China; ^8^ Department of Biomedicine University of Bergen Jonas Lies vei 91 Bergen Norway 5009

**Keywords:** astrocytes, Glioma, myeloid inflammatory cells, radiation‐induced senescence, senolytic agents

## Abstract

Accumulating evidence suggests that changes in the tumor microenvironment caused by radiotherapy are closely related to the recurrence of glioma. However, the mechanisms by which such radiation‐induced changes are involved in tumor regrowth have not yet been fully investigated. In the present study, how cranial irradiation‐induced senescence in non‐neoplastic brain cells contributes to glioma progression is explored. It is observed that senescent brain cells facilitated tumor regrowth by enhancing the peripheral recruitment of myeloid inflammatory cells in glioblastoma. Further, it is identified that astrocytes are one of the most susceptible senescent populations and that they promoted chemokine secretion in glioma cells via the senescence‐associated secretory phenotype. By using senolytic agents after radiotherapy to eliminate these senescent cells substantially prolonged survival time in preclinical models. The findings suggest the tumor‐promoting role of senescent astrocytes in the irradiated glioma microenvironment and emphasize the translational relevance of senolytic agents for enhancing the efficacy of radiotherapy in gliomas.

## Graphical abstract

Senescent astrocytes (SnAs) induced by Ionizing radiation (IR) modulate the secretory profiles of GBM cells via senescence‐associated secretory phenotype (SASP) to remodel the tumor immue microenvironment and promote GBM recurrence. Selectively clearance of these SnAs by senolytic drugs can delay tumor growth.

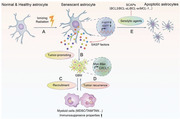

## 1 Introduction

In adults, glioblastoma (GBM) is the most lethal type of primary brain tumor.^[^ ^1^ ^]^ This tumor's response to postoperative treatment, including radiotherapy (RT) combined with temozolomide chemotherapy, is often transient, with tumors invariably recurring within a relatively short period.^[^ ^2^ ^]^ Most cases of GBM recurrences occur within the previous RT field, exhibiting more invasive and therapy‐resistant phenotypes.^[^ ^3^ ^]^ Studies have consistently reported that radiation‐induced changes in the tumor microenvironment may play an important role in the pattern of GBM regrowth, despite the tumor cell‐intrinsic mechanism of resistance.^[^ ^4^ ^]^

Ionizing radiation (IR) is a widely used cancer therapy that has dual effects on the tumor immune microenvironment (TIME).^[^ ^4^, ^5^ ^]^ RT not only eliminates residual tumor cells but also has the potential to initiate systemic antitumor immune responses by facilitating the release of tumor antigens and immune effector molecules. However, it also promotes immune evasion of tumor cells. By increasing the expression of several immunosuppressive cytokines and chemokines, RT induced a great amount of myeloid inflammatory cells to migrate to residual tumor areas, thereby accelerating vascular remodeling and DNA damage repair to promote tumor recurrence.^[^ ^6^ ^]^

In the context of cancer, cellular senescence is an irreversible state of cell cycle arrest that is usually caused by therapeutic interventions (RT and chemotherapy) or oncogene activation.^[^ ^7^ ^]^ Although therapy‐induced senescence (TIS) was initially considered a tumor suppressive mechanism, at present, it is well‐known that senescent stromal cells can support neighboring tumor cells in a paracrine‐dependent manner.^[^ ^8^ ^]^ Senescent cells (SnCs) exhibit several distinct features, including activation of cell cycle inhibitory pathways such as the p53/p21^CIP1^ and p16^INK4A^/pRB pathways, increased senescence‐associated β‐galactosidase (SA‐β‐Gal) activity, and an altered expression profile, called senescence‐associated secretory phenotype (SASP).^[^ ^7^ ^]^ The entire SASP profile comprises various soluble factors, including proinflammatory cytokines, chemokines, growth factors, and extracellular matrix components or metalloproteases.^[^ ^9^ ^]^ Several studies have reported that SASP‐related cytokines, such as IL‐6, IL‐8, CCL2, TNF‐α, and TGF‐β, can induce chronic inflammation by recruiting immunosuppressive cells.^[^ ^10^ ^]^ However, information about the precise mechanism by which these SASP factors mediate suppressive TIME formation after irradiation is scarce.

A novel “one‐two punch” cancer therapy concept has emerged that senolytic agents can be used as an adjuvant therapy after traditional treatment to target TIS.^[^ ^8a^ ^]^ Preclinical studies have reported that combination therapy is an effective treatment strategy for many cancer types.^[^ ^11^ ^]^ Apart from preventing tumor relapse and reprogression, the selective clearance of these SnCs can alleviate therapy‐induced side effects.^[^ ^12^ ^]^ Using IR to induce senescence in the brain microenvironment, we observed that astrocytes, the most abundant SnC type, are enough to promote the progression of glioma by recruiting myeloid inflammatory cells to the TIME. Furthermore, the SASP of senescent astrocytes (SnAs) increased chemokine secretion from GBM cells, such as CXCL1. Senolytic drugs such as ABT263 or dasatinib plus quercetin (D+Q) combined with RT can significantly prolong the median survival time of tumor‐bearing mice. Taken together, we elucidated the possible mechanism by which SnAs contribute to GBM regrowth after RT by inducing a TIME and provided new insights for improving the existing treatment strategies for glioma to delay tumor recurrence.

## 2 Results

### 2.1 IR‐Induced Accumulation of SnCs in the Brain Accelerates Tumor Growth

**Figure 1:**
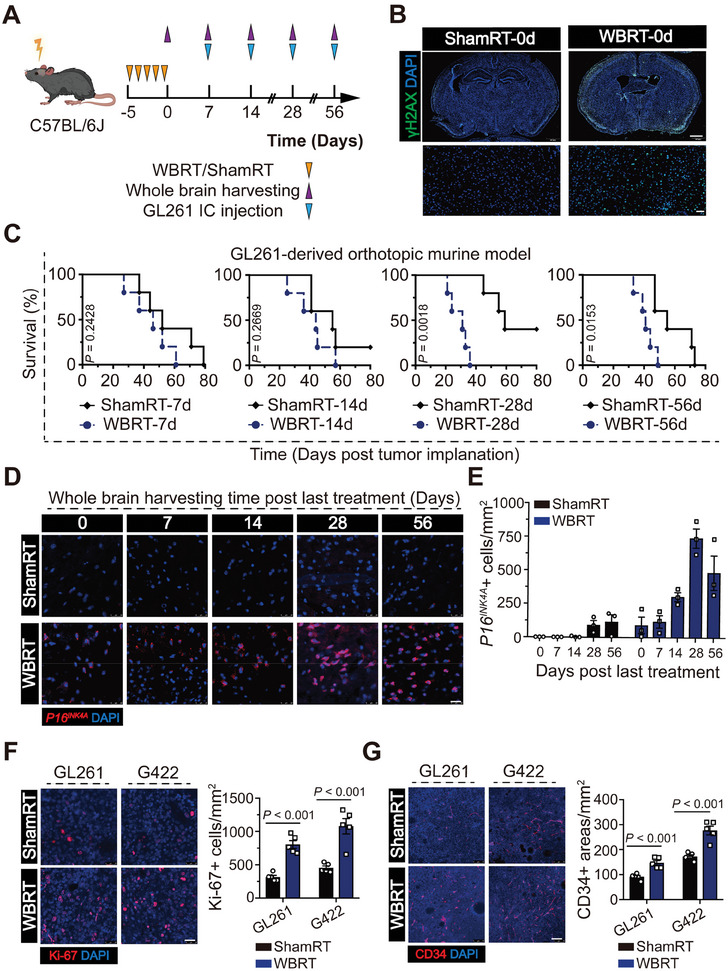
SnCs accumulate in the brain after ionizing radiation (IR) and promote glioma progression. A) Schematic representation of the experimental design. B) Representative immunofluorescence images showing the γH2AX foci (green) in the DAPI‐stained nuclei (blue) of irradiated or mock‐irradiated mouse brains. Brains were harvested and fixed at 2 hours after the final IR. Scale bars, 1000 µm (upper) and 50 µm (lower). C) Kaplan–Meier graphs showing the survival time of irradiated or mock‐irradiated mice with orthotopic implantation of GL261 xenografts at indicated time points after IR (*n* = 5 per group); log‐rank test. D,E) Representative images (D) and quantification (E) of *P16^INK4A^* RNA FISH of mouse brains at indicated time points after IR. Scale bar, 25 µm. F) Representative images and quantification of Ki‐67 immunofluorescence staining of GL261‐ and G422‐derived xenografts. Scale bar, 25 µm. G) Representative images and quantification of CD34 immunofluorescence staining of GL261‐ and G422‐derived xenografts. Scale bar, 75 µm.

To determine whether IR‐induced changes in the brain affect GBM growth in vivo, whole brain of immunocompetent C57BL/6J mice were preirradiated at 0 or 2 Gy for five consecutive days, followed by intracranial implantation of a limited number of GL261 cells. Mouse brains were harvested at 7, 14, 28, and 56 days post IR (**Figure** **1**A). The γH2AX foci were dramatically increased post the final IR of 2 Gy, indicating that extensive DNA damage was induced (Figure 1B). Additionally, we found that the survival time of tumor‐bearing mice was obviously shorter in the preirradiated mice group than in the mock‐irradiated mice group, particularly at 28 days after IR (GL261: 46 days vs 52 days, WBRT‐7d vs ShamRT‐7d, respectively, *P* = 0.2428; 44 days vs 55 days, WBRT‐14d vs ShamRT‐14d, respectively, *P* = 0.2669; 31 days vs 59 days, WBRT‐28d vs ShamRT‐28d, respectively, *P* = 0.0018; 41 days vs 55 days, WBRT‐56d vs ShamRT‐56d, respectively, *P* = 0.0153; Figure 1C). To validate IR‐induced senescence in the brain, we subjected mouse brains that were mock‐irradiated or irradiated at 7, 14, 28, and 56 days after IR to RNA FISH using *P16^INK4A^*, a widely used senescence marker. The expression of *P16^INK4A^* was increased in the pre‐irradiated brains, with expression peaking at 28 days post IR (Figure 1D,E; Figure S1A, Supporting Information). Subsequently, we repeated the experiment with another synergetic mouse GBM cell line‐G422 cells. Preirradiated mice bearing GL261 or G422 cells exhibited a shorter survival time than mock‐irradiated group (GL261: 33 days vs 59.5 days, WBRT‐28d vs ShamRT‐28d, respectively, *P* = 0.0051; G422: 29.5 days vs 44.5 days, WBRT‐28d vs ShamRT‐28d, respectively, *P* = 0.0066; Figure S1B, Supporting Information). In addition, significantly increased microvascular proliferation and Ki‐67+ proliferating cells was also observed in tumors developed in preirradiated mice, indicating a more aggressive growth pattern (Figure 1F,G; Figure S1C, Supporting Information). However, although a single 10 Gy dose of IR can trigger cellular senescence in RBOs at 7 days after IR, the tumor‐promoting effects of sen‐RBO were not significant compared with those of Nor‐RBOs in the GBM‐RBO coculture system in vitro; this indicates peripheral immune cells might be involved (Figure S2, Supporting Information). Taken together, these findings suggest that IR‐induced SnCs accumulation has potent tumor‐promoting effects.

### 2.2 Pharmacogenetic Clearance of SnCs Prolongs the Survival Time of Preirradiated Mice Bearing GBM Cells

**Figure 2:**
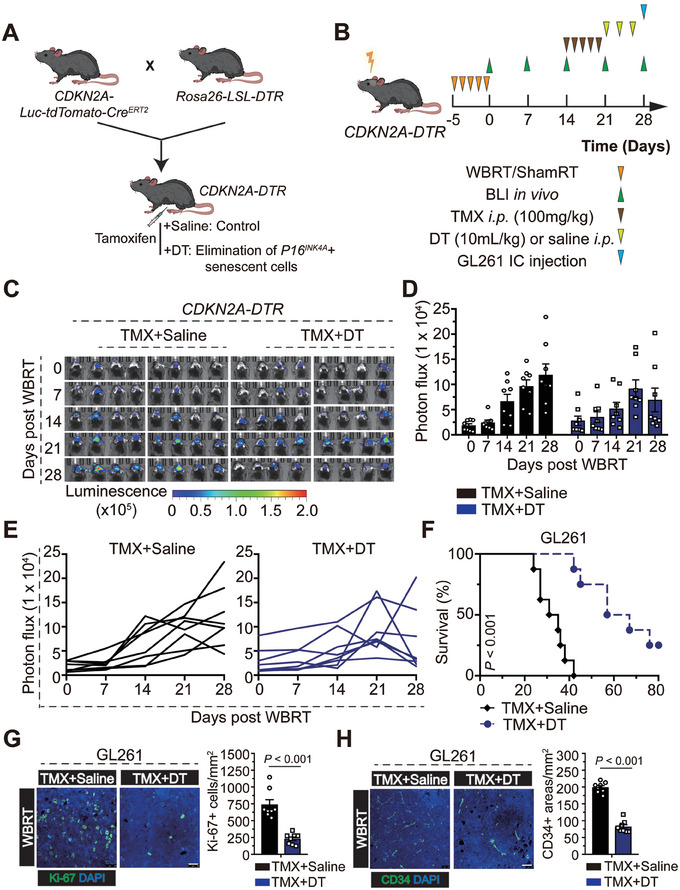
Pharmacogenetic approach prolongs the survival time of pre‐irradiated *CDKN2A*‐DTR mice bearing GL261 cells. A) *CDKN2A*‐DTR mice were generated by crossing *Rosa26‐LSL‐iDTR* with *CDKN2A‐Luc‐tdTomato‐Cre^ERT2^*. Mice were injected tamoxifen for consecutive 5 days at 14 days after the final IR, followed by the administration of diphtheria toxin for another 3 days to selectively eliminate *P16^INK4A^* + SnCs. B) Schematic representation of the experimental design. C–E) Representative images (C) and quantification (D,E) of the in vivo bioluminescence imaging of *CDKN2A*‐DTR mice at the indicated time points after IR. F) Kaplan–Meier graph showing the survival time of pre‐irradiated *CDKN2A*‐DTR mice bearing GL261 cells (*n* = 8 per group); log‐rank test. G) Representative images and quantification of Ki‐67 immunofluorescence staining from GL261‐derived xenografts in *CDKN2A*‐DTR mice. Scale bar, 25 µm. H) Representative images and quantification of CD34 immunofluorescence staining from GL261‐derived xenografts *in CDKN2A*‐DTR mice. Scale bar, 25 µm.

To elucidate the in vivo role of *P16^INK4A^* + SnCs in GBM progression, we generated a *CDKN2A‐DTR* (*CDKN2A‐Luc‐tdTomato‐Cre^ERT2^+/‐; Rosa26‐LSL‐iDTR+/‐*) mouse model to identify, track, and selectively kill *P16^INK4A^* + SnCs in vivo (**Figure** **2**A,B). Using in vivo bioluminescence, exposure of *CDKN2A‐DTR* mice to IR induced a time‐dependent increase in the abundance of SnCs in the brains within 28 days, while TMX plus DT selectively eliminated these SnCs (Figure 2C–E). Clearance of *P16^INK4A^* + SnCs extends the survival time of preirradiated mice bearing GL261 cells (GL261: 62 days versus 33 days, TMX+DT versus TMX+Saline, respectively, *P* < 0.001; Figure 2F), accompanied by decreased microvascular and cellular proliferation in the tumor area (Figure 2G,H). These data suggest that SnCs clearance alleviates IR‐induced changes and associated GBM growth in vivo.

### 2.3 Astrocytes are the Most Predominant Senescent Subpopulation Triggered by IR in the Brain and Exhibit Changes in Secretory Phenotypes

**Figure 3:**
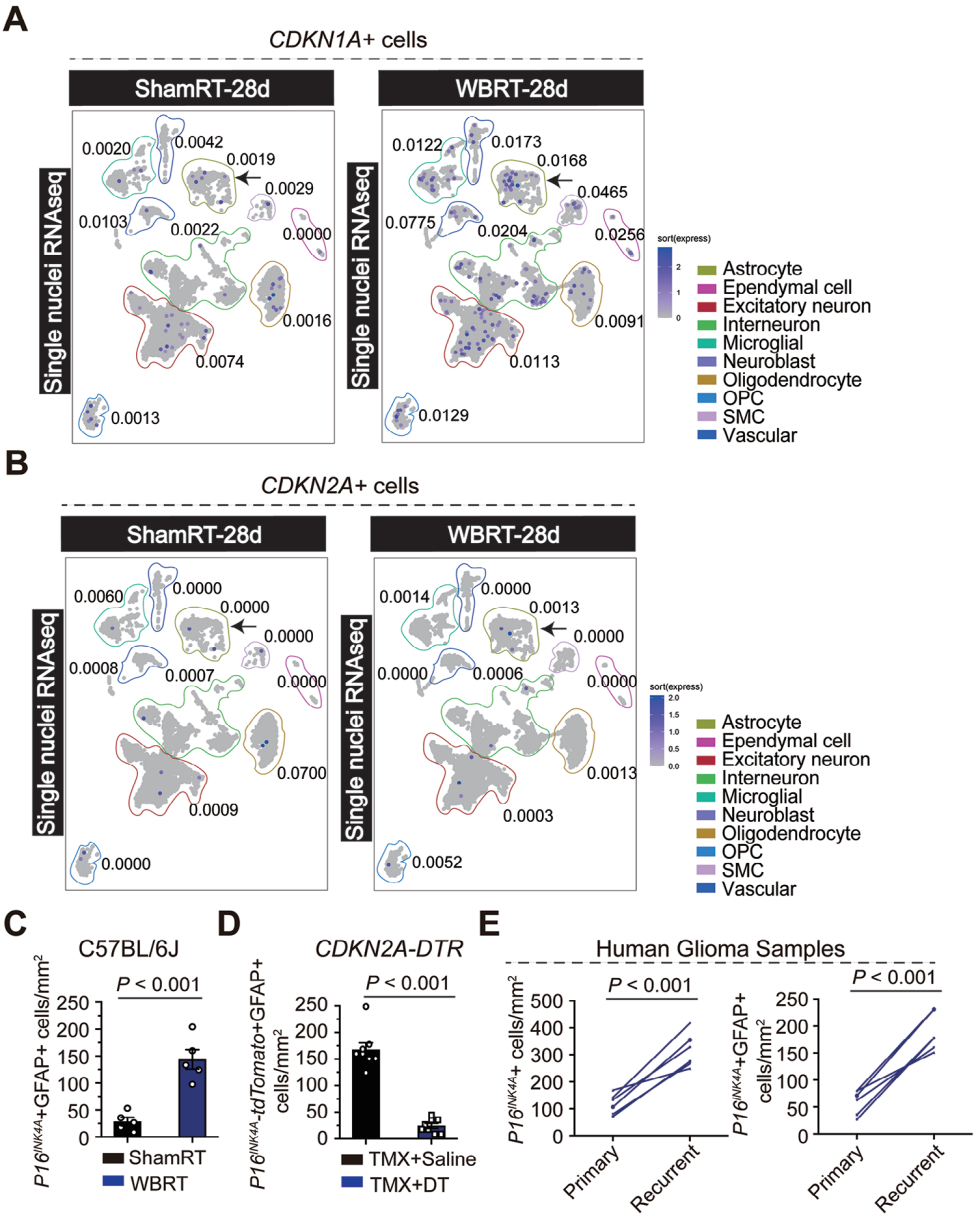
IR triggers the most prominent increase in senescent astrocyte population in the brain. A,B) UMAP embeddings with the expression of *P21^CIP1^* (A) or *P16^INK4A^* (B) for snRNA‐seq datasets. Cell type annotations were represented as indicated color frames. The percentage of cells positive for high levels of *P16^INK4A^* or *P21^CIP1^* is indicated next to each cell population. C) Quantification of *P16^INK4A^* and GFAP in the brain sections of wild‐type C57BL6/J mice via immune‐RNA FISH. D) Quantification of *P16^INK4A^*‐tdTomato‐positive astrocytes (GFAP^+^) in the brain sections of *CDKN2A*‐DTR mice. E) Quantification of *P16^INK4A^* and GFAP in paired primary and recurrent glioma samples (*n* = 12) via immune‐RNA FISH.

Previous studies have reported that IR can trigger the cellular senescence of non‐neoplastic cells. However, the identity of these SnCs remains unclear because most of these studies used semiquantitative methods such as RNA FISH, immunofluorescence analysis, or IHC analysis, and therefore, lacked a comprehensive understanding of senescence in different cell types.^[^ ^13^ ^]^ In the present study, we performed snRNA‐seq to profile and compare the cellular composition and transcriptomes of irradiated (WBRT‐28d) and mock‐irradiated (ShamRT‐28d) mouse brains (Supplementary Figure S3A). By measuring the expression of *P16^INK4A^* and *P21^CIP1^*, two commonly used markers for senescence, we observed that IR increased number of *P16^INK4A^+* and *P21^CIP1^+* cells, with the most prominent and uniform increase in astrocyte and oligodendrocyte progenitor cell (OPC) populations (**Figure** **3**A,B). In addition, the increased number and percent of SnCs were very similar between astrocyte and OPC populations; nevertheless, the total number of SnCs was higher in the astrocytic population (Figure 3A,B; Figure S3B, Supporting Information). Next, we analyzed the expression of cytokines and growth factors, also known as SASP factors, in the abovementioned cell populations.^[^ ^14^ ^]^ Of note, the astrocytic population exhibited the highest expression of the listed SASP factors (Figure S3C, Supporting Information). Further, we also observed that the fraction of positive cells for *Nrg1*, *Trp53*, *Map2k6*, *Prodh*, *Hmgb1*, and *Col19a1* was increased in the WBRT‐28d group (Figure S3D, Supporting Information). To further determine the identity of the SnCs types induced by IR, we assessed in vivo *P16^INK4A^* expression using RNA FISH with astrocyte markers. We found the number of *P16^INK4A^‐*positive astrocytes increased dramatically, not only in mouse brains of the WBRT group, but also in recurrent glioma samples compared to paired primary samples (Figure 3C–E; Figure S3E,F,G, Supporting Information). We also assessed these widely known SASP factors in OPC subpopulation. Surprisingly, we found that the fractions of positive cells for *Mmp14*, *Cxcl12*, *Trp53*, *Brf1*, *Hgf*, *Igfbp3*, *Prodh*, *Il‐18*, and *Mif*, which were predominantly expressed in OPCs (Supplementary Figure S3C), exhibited no statistical difference comparing ShamRT‐28d with WBRT‐28d. These results from astrocytic and OPC subpopulations presumably indicated distinct mechanisms underlying the tumor‐promoting function of those two types of senescent cells (Figure S3H, Supporting Information).

**Figure 4:**
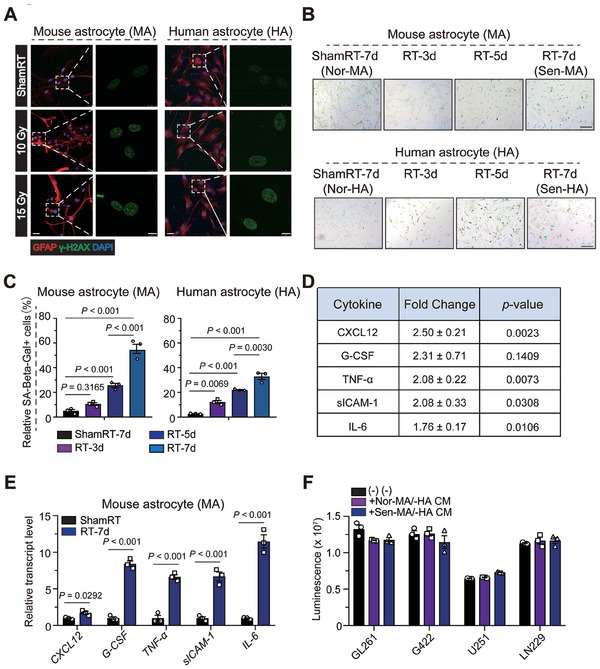
Senescent astrocytes exhibit changes in secretory cytokine profile. A) Representative images of GFAP and γH2AX immunofluorescence costaining in irradiated or mock‐irradiated MA and HA at 2 h after IR. Scale bars, 25 and 10 µm. B) Representative images of senescence‐associated β‐galactosidase staining in irradiated or mock‐irradiated MA and HA at indicated time points after IR. Scale bar, 200 µm. C) Quantification of senescence‐associated β‐galactosidase staining of irradiated or mock‐irradiated mouse astrocytes (MA) and human astrocytes (HA) at indicated time points after IR. D) MA were irradiated or mock‐irradiated; after 7 days, conditioned medium (CM) was collected and subjected to multiple cytokine array analysis. Fold increase and indicated *p*‐values of the irradiated and mock‐irradiated groups are shown in the table. Experiments were performed in triplicate. E) *mRNA* was extracted and subjected to qRT‐PCR. *β‐actin* was used as internal control. F) GBM cells (GL261, G422, U251, and LN229) were incubated with the indicated MA/HA‐CM for 72 h and subjected to CellTiter Glo assay.

To elucidate the effects and possible molecular mechanism of SnCs on GBM progression, we performed cytokine analysis using CM derived from normal astrocytes and SnAs (**Figure** **4**A,B,C). Of the 40 cytokines that were screened, only eighteen cytokines were present at detectable level, and the levels of five cytokines were significantly increased (Figure 4D; Figure S4A, Supporting Information). Further, the mRNA expressions of *CXCL12, G‐CSF, TNF‐α, sICAM‐1*, and *IL‐6* mRNA were also found increased in SnAs compared to normal astrocytes (Figure 4E). However, incubation with SnAs‐CM did not significantly alter the proliferation of GBM cells in vitro; this result is inconsistent with in vivo data, suggesting indirect tumor‐promoting role of SASP factors derived from SnAs (Figure 4F). Altogether, these results suggest that the most prominent increase of SnCs induced by IR is the astrocytic population, with substantial changes in secretory phenotypes.

### 2.4 TNF‐α Derived from SnAs Activate the Myc‐Max Signaling Pathway to Promote CXCL1 Production in GBM Cells

**Figure 5:**
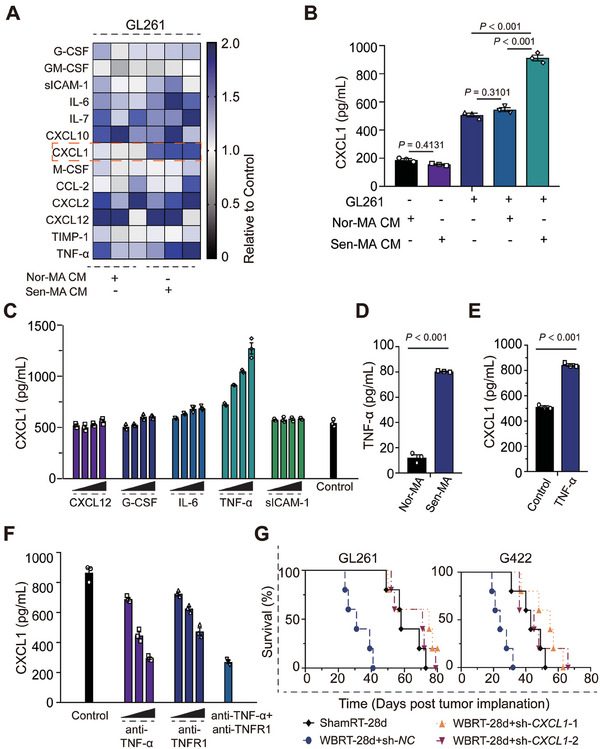
TNF‐α derived from senescent astrocytes drives the production of immunosuppressive cytokines in GBM cells. A) GL261 cells were incubated with Control‐/Nor‐MA‐/Sen‐MA‐CM for 48 h; then, supernatants were collected and subjected to multiplex cytokine array analysis. The fold increase of Nor‐/Sen‐MA‐CM groups over that of the control is shown in the heatmap. B) CXCL1‐ELISA revealed the stimulation of CXCL1 production in GL261 cells by incubation with Sen‐MA‐CM. CXCL1 levels in Nor‐MA/Sen‐MA‐CM were assessed and served as controls. C) GL261 cells were incubated with differentially expressed cytokines from Sen‐MA‐CM and compared with Nor‐MA‐CM (CXCL12, G‐CSF, TNF‐α, sICAM‐1 and IL‐6) at gradient concentrations (0, 50, 250, 500, and 1000 pg mL^−1^) for 24 h. The supernatants were collected and subjected to CXCL1‐ELISA. D) ELISA showing increased TNF‐α level in Sen‐MA‐CM relative to Nor‐MA‐CM. E) CXCL1 production in GL261 cells was increased following TNF‐α treatment at the concentration detected in Sen‐MA‐CM (80 pg mL^−1^). F) Increased CXCL1 production induced by TNF‐α was reduced following treatment with TNF‐α‐ or TNFR1‐neutralizing antibodies. G) Kaplan–Meier graph showing the survival time of pre‐irradiated mice bearing GL261‐sh‐*NC* or ‐sh‐*CXCL1*‐1/2 cells (*n* = 5 per group); log‐rank test.

Next, we investigated the mechanisms by which secretory phenotypes of SnAs promote GBM progression, with initially focusing on immunosuppressive characteristics. GL261 cells were incubated with Nor‐MA‐ or Sen‐MA‐CM, and the supernatants were collected for multiplex cytokine array analysis. Among the 40 cytokines that were screened, CXCL1 was the most actively upregulated cytokine; it plays an essential role in recruiting CXCR2‐positive myeloid inflammatory cells, including neutrophils, monocytes and macrophages, and inducing local angiogenesis (**Figure** **5**A,B; Figure S5A,B,C, Supporting Information).^[^ ^15^ ^]^ To identify the cytokines secreted by SnAs that were responsible for increased CXCL1 production in GBM cells, we treated GL261 cells with these cytokines (gradient increase in concentrations) and tested CXCL1 levels via ELISA. Among the five cytokines, G‐CSF, IL‐6, and TNF‐α were able to elevate CXCL1 production; TNF‐α had the most prominent effects (Figure 5C). Similar results were obtained using the same concentration of TNF‐α measured in SnAs‐CM (Figure 5D,E; Figure S5D, Supporting Information). Further, neither TNFR1 inhibition nor TNF‐α neutralization reduced CXCL1 production in GL261 cells (Figure 5F). To explore the biological role of CXCL1 in GBM progression, GL261/G422‐sh‐*CXCL1* cell lines were established and implanted into pre‐irradiated mouse brains. Knockdown of *CXCL1* in GBM cells abrogated the protumorigenic effects of SnAs and extended the survival time of pre‐irradiated mice bearing GL261 or G422 cells (GL261: 75 or 71 days vs 31 days, WBRT+sh‐*CXCL1*‐1/2 vs WBRT+sh‐NC, respectively, *P* < 0.01; G422: 55 or 45 days vs 24 days, WBRT+sh‐*CXCL1*‐1/2 vs WBRT+sh‐NC, respectively, *P* < 0.01; Figure 5G). Collectively, these results suggest that TNF‐α derived from SnAs is the cytokine responsible for inducing CXCL1 production in GBM cells to promote GBM progression.

**Figure 6:**
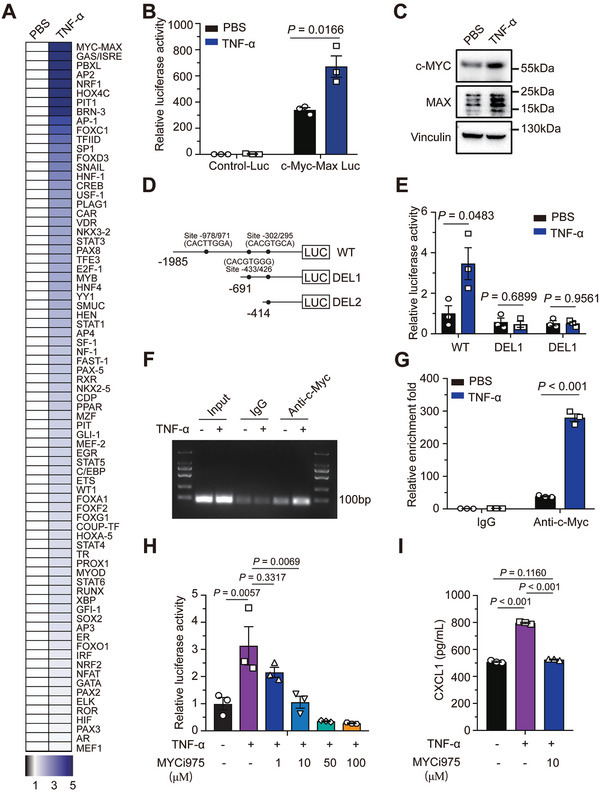
TNF‐α derived from senescent astrocytes activates Myc‐Max signaling in GBM cells. A) GL261 cells were treated with TNF‐α (80 pg mL^−1^) for 72 h. Nuclei proteins were extracted and subjected to multiplex profiling analysis for transcriptional activation. The activity of each transcriptional factor was normalized to that of the PBS‐treated group. B) GL261 cells were transfected with control or c‐Myc‐Max promoter *firefly* luciferase constructs along with *renilla* luciferase reporters, followed by TNF‐α treatment for 48 h. *Firefly* luciferase activity was normalized by *renilla* luciferase activity and compared with the control group. C) Western blot analysis of c‐Myc and Max proteins in lysates prepared from GL261 cells. D) Schematic diagram of the *firefly* luciferase constructs of a 1985‐bp region upstream of CXCL1 TSS and indicated truncates designed by predicting binding sites of c‐Myc. E) GL261 cells were transfected with constructs in (D) and *renilla* reporter, followed by TNF‐α treatment for 48 h. *Firefly* luciferase activity was normalized by *renilla* luciferase activity and compared with the control group. F,G) Nuclear extracts were subjected to chromatin immunoprecipitation assay. Immunoprecipitants analyzed by PCR and electrophoresis (F) and qRT‐PCR (G) showing elevated fold enrichment of the promoter site −978/971 in the anti‐c‐Myc group. H) GL261 cells were transfected with control or c‐Myc‐Max promoter *firefly* luciferase constructs along with *renilla* luciferase reporters, followed by TNF‐α or TNF‐α plus MYCi975 treatment for 48 h. *Firefly* luciferase activity was normalized by *renilla* luciferase activity and compared with the control group. (I) CXCL1 production induced by TNF‐α was reduced after coadministration with MYCi975 measured by ELISA.

To explore the mechanism by which TNF‐α stimulated the expression of *CXCL1*, we evaluated DNA‐binding activity of 96 transcriptional factors in GL261 cells using a multiplex screening assay kit. TNF‐α treatment activated multiple transcriptional factors, including Myc‐Max, GAS/ISRE, PBXL, and AP2 (**Figure** **6**A). The upregulated transcriptional activity of Myc‐Max was further verified using dual luciferase reporter assay (Figure 6B; Figure S5E,F, Supporting Information), while protein levels of c‐Myc and Max were also increased in response to TNF‐α (Figure 6C). Next, we analyzed the promoter sequence of *CXCL1* and predicted the possible binding regions of c‐Myc using the motif recognition method. We observed that TNF‐α increased the binding ability of c‐Myc to the −978/971 region of *CXCL1* promoter; this result was further validated using the luciferase reporter assay and ChIP assays (Figure 6D,E,F,G). Disruption of Myc/Max complex and inhibition of their transcriptional activity by MYCi975 alleviated TNF‐α‐induced CXCL1 production in GL261 cells (Figure 6H,I).^[^ ^16^ ^]^ Taken together, our results suggest that TNF‐α secreted by SnAs promotes CXCL1 production by activating the transcriptional activity of c‐Myc‐Max in GBM cells.

### 2.5 TIME is Remodeled by Pharmacological Clearance of SnCs and the Survival Time of Tumor‐Bearing Mice is Extended

**Figure 7:**
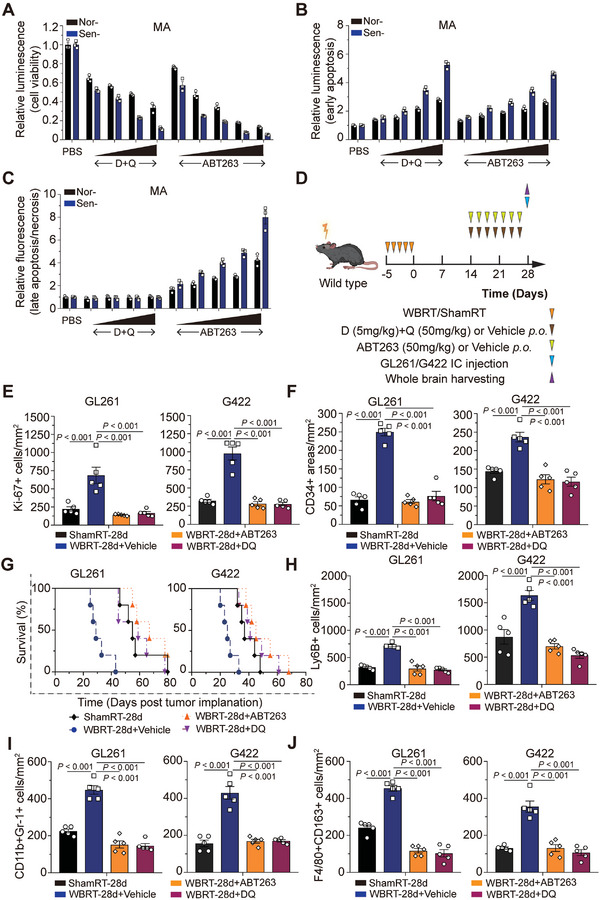
Tumor immune microenvironment is remodeled by clearance of senescent cells using senolytic drugs in vivo. A) Nor‐/Sen‐MA were incubated with gradient concentrations of ABT263 (1, 5, 20, 50, 100 µM) or D+Q (1/20, 10/20, 10/40, 20/40 µm) for 72 h and subjected to CellTiter Glo assay. B,C) Nor‐/Sen‐MA were incubated with gradient concentrations of ABT263 (1, 5, 20, 50, 100 µm) or D+Q (1/20, 10/20, 10/40, 20/40 µm) for 24 h and subjected to RealTime‐Glo Annexin V Apoptosis Assay. D) Schematic representation of the experimental design. E,F) Quantification of Ki‐67 (E) and CD34 (F) immunofluorescence staining from GL261‐ and G422‐derived xenografts. G) Kaplan–Meier graph showing the survival time of preirradiated mice bearing GL261 cells administrated with ABT263 or D+Q (*n* = 5 per group); log‐rank test. H) Quantification of Ly6B immunofluorescence staining of GL261‐ and G422‐derived xenografts. Scale bar, 25 µm. I) Quantification of immunofluorescence costaining against CD11b and Gr‐1 of GL261‐ and G422‐derived xenografts. Scale bar, 25 µm. J) Quantification of immunofluorescence costaining against F4/80 and CD163 from GL261‐ and G422‐derived xenografts. Scale bar, 25 µm.

SnCs exhibit high antiapoptotic effects by upregulating the expression of *BCL‐2*, *BCL‐xL*, *BCL‐w*, and *MCL‐1* or activating the prosurvival signaling pathways such as PI3K/AKT to promote cellular survival. In our study, we observed that the expression of *BCL‐2*, *MCL‐1*, and *BCL‐w* was significantly elevated in the astrocytes of irradiated mice compared to those of mock‐irradiated mice (Figure S6A, Supporting Information). Further, increased protein levels of BCL‐2, BCL‐w, and BCL‐xL and phosphorylation of AKT was verified by WB (Figure S6B, Supporting Information). Based on these findings, we chose ABT263 and D+Q for subsequent experiments because they have exhibited the ability to clear SnCs in mouse models of neurodegenerative diseases in previous studies (Figure S6C, Supporting Information). Interestingly, both ABT263 and D+Q significantly and dose‐dependently inhibited cell viability of sen‐MA or ‐HA (**Figure** **7**A; Figure S7A, Supporting Information). D+Q mainly induced early apoptosis, whereas ABT263 promoted late apoptosis or necrosis at 24 h post incubation, indicating a faster kinetics of ABT263 compared with D+Q (Figure 7B,C; Figure S7B,C, Supporting Information). Moreover, oral administration of ABT263 or D+Q via gavage significantly eliminated SnCs in preirradiated mouse brains, thereby delaying tumor growth (Figure 7D,E,F,G; Figures S7D,E, and S8A, Supporting Information; GL261: 55 days or 67 days or 59 days vs 29 days, ShamRT‐28d or WBRT‐28d+ABT263 or WBRT‐28d+DQ vs WBRT‐28d+Vehicle, respectively, *P* < 0.01; G422: 37 days or 45 days or 41 days vs 25 days, ShamRT‐28d or WBRT‐28d+ABT263 or WBRT‐28d+DQ vs WBRT‐28d+Vehicle, respectively, *P* < 0.01). This further enhanced the recruitment of myeloid inflammatory cells, including neutrophils (Ly6B^+^), MDSCs (CD11b^+^Gr‐1^+^), and macrophages (F4/80^+^CD163^+^), in pre‐irradiated mouse brains bearing tumor; this recruitment was uniformly decreased in response to ABT263 or D+Q treatment or knockdown of *CXCL1* (Figure 7H,I,J; Figures S8B,C,D and S9A,B,C, Supporting Information).

**Figure 8:**
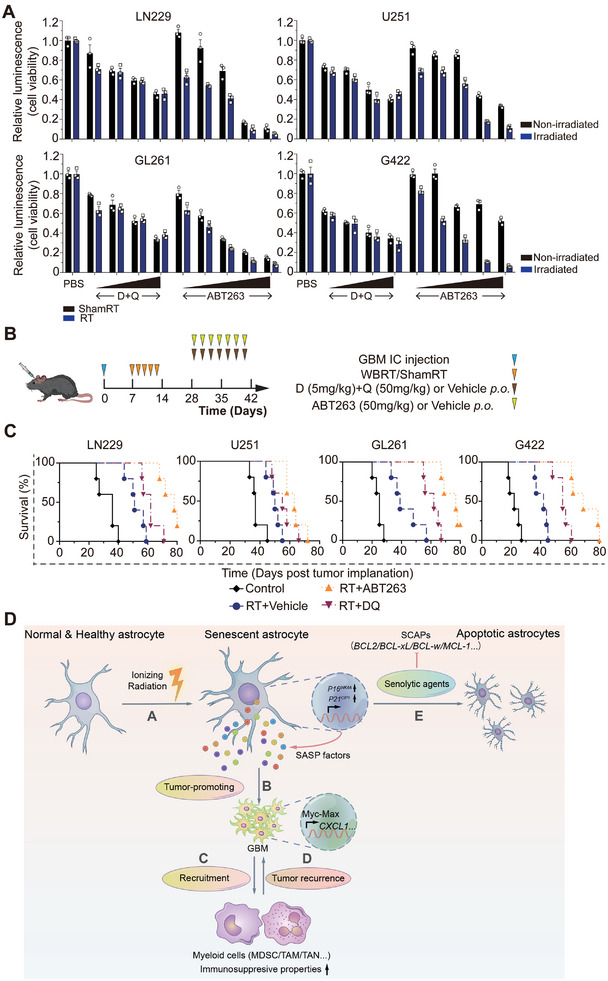
Senolytic drugs help enhance the efficacy of radiotherapy. A) Irradiated and mock‐irradiated GBM cells were incubated with gradient concentrations of ABT263 (1, 5, 20, 50, 100 µm) or D+Q (1/20, 10/20, 10/40, 20/40 µm) for 72 h and subjected to CellTiter Glo assay. B) Schematic representation of the experimental design. C) Kaplan–Meier graph showing the survival time of tumor‐bearing mice with indicated treatment (*n* = 5 per group); log‐rank test. D) Schematic model of the mechanism. Senescent astrocytes (SnAs) induced by IR modulate the secretory profiles of GBM cells via SASP to remodel the tumor immue microenvironment and promote GBM recurrence. DNA damage is induced by IR in normal astrocytes in the peritumoral regions. These cells undergo cellular senescence by upregulating cell cycle inhibitors such as *P16^INK4A^* and *P21^CIP1^*, followed by the release of a large amount of SASP factors. SnAs‐derived soluble cytokines, such as TNF‐α, activates downstream Myc‐Max signaling and induces transcription of *CXCL1* in GBM cells, which is responsible for the recruitment of myeloid inflammatory cells, in turn leading to tumor recurrence. Selectively clearance of these SnAs by senolytic drugs can delay tumor growth.

To determine the therapeutic efficacy of IR in combination with senolytic drugs for GBM treatment, we first evaluated the cell viability of irradiated or mock‐irradiated GBM cells in response to ABT263 or D+Q by CellTiter‐Glo assay. We observed that ABT263 inhibited the proliferation of irradiated GBM cells but had mildly inhibitory effects in those mock‐irradiated; while D+Q had limited effects on GBM cells no matter they were irradiated or mock‐irradiated (**Figure** **8**A). Furthermore, oral administration of ABT263 or D+Q after IR in tumor‐bearing mice significantly extended their survival time (GL261: 77 days or 62 days vs 39 days, RT+ABT263 or RT+DQ vs RT+Vehicle, respectively, *P* < 0.01; G422: 69 days or 55 days vs 42 days, RT+ABT263 or RT+DQ vs RT+Vehicle, respectively, *P* < 0.01; U251: 63 days or 55 days vs 50 days, RT+ABT263 or RT+DQ vs RT+Vehicle, respectively, *P* < 0.05; LN229: 78 days or 62 days vs 51 days, RT+ABT263 or RT+DQ vs RT+Vehicle, respectively, *P* < 0.01; Figure 8B,C). However, divergences of therapeutic efficacy were observed between different cell types, which remains to be investigated in the future. Taken together, these results suggest that selective clearance of SnCs by senolytic drugs after IR can delay tumor recurrence, highlighting the possibility of using senolytic therapy as an additional treatment regimen for GBM.

## 3 Discussion

GBM is the most common and malignant primary brain tumor with the poorest prognosis.^[^ ^1^ ^]^ RT, as an important aspect of current clinical treatment strategies against glioma, can locally control tumor progression in a relatively short time, with a progression‐free survival of only 6–9 months.^[^ ^2b^ ^]^ Previous studies mainly focused on the mechanisms by which tumor cell adaption resulted in resistance to RT, such as autophagy, GSC maintenance, and hypoxic response; however neglected the remodeling effects of RT on the tumor microenvironment. Gliomas often recur in the peritumoral brain area that receives high‐dose radiation; this suggests that stromal cells respond to radiation in ways that can be advantageous to GBM cells.^[^ ^3^, ^4^, ^17^ ^]^ However, changes in the microenvironment caused by RT and the underlying mechanisms affecting tumor recurrence remain unelucidated.

Recent studies have reported that IR‐induced senescent stromal cells can support neighboring tumor cells via paracrine way.^[^ ^11^, ^18^ ^]^ To determine the relationship between SnCs accumulation and GBM recurrence, we preirradiated mouse brains followed by IC injection of GBM cells at different time points. We found that as the time increased, SnCs generally accumulated in the brain, peaking at 28 days after IR; whereas still sustained at relative high level at 56 days after IR. It has been well‐known that SASP can trigger senescence of the surrounding normal cells, also termed at paractine senescence, which has been shown to drive the accumulation of SnCs.^[^ ^19^ ^]^ Meanwhile, immunosurveillance is one of the most crucial processes to remove cells undergoing senescence.^[^ ^20^ ^]^ In addition, the survival time of preirradiated mice bearing GBM cells was shortened accordingly. These results support the emerging view that stromal cells may respond to radiation such that they possibly prime the tumor microenvironment for GBM recurrence, such as TIS.^[^ ^6^, ^17^ ^]^ Interestingly, using snRNA‐seq, we observed that several kinds of cell types underwent senescence in the brain after IR, including astrocytes and OPCs. The astrocytic population was chosen for our subsequent studies as they are the most abundant cell type in the brain; further, they have been reported to be prone to senescence, thereby contributing to neurodegenerative diseases or glioma progression.^[^ ^11^, ^13^, ^21^ ^]^ These results were corroborated by analyzing tissue samples from recurrent human gliomas after chemoradiation; the abundance of SnAs increased compared with that of patient‐matched treatment‐naive gliomas. In addition, the actual number of astrocytes increased possibly owing to transition from one kind of astrocyte state to another, i.e., astrogliosis in the early stage after IR and astrocyte senescence in the late stage.^[^ ^21a^ ^]^ Nevertheless, how the cellular states of astrocytes were modulated and transitioned between each other remains unclear. Furthermore, the effects of other kinds of senescent cell types on glioma progression also need to be investigated, such as OPCs. Senescence of OPCs could lead to cognitive impairment diseases such as Alzheimer's disease (AD).^[^ ^22^ ^]^ However, the function of OPCs within TME has been underestimated for a very long time. Studies investigating therapy‐induced senescent OPCs and their effects on tumor progression are rare. Surprisingly, we examined well‐known SASP factors in OPC subpopulation and found distinct secretory profiles compared with astrocytic subpopulation, possibly due to their actions in myelination. Hide et al have reported the phenomenon of accumulation of OPCs at peritumoral regions in human gliomas. OPCs‐derived cytokines, such as EGF and FGF1, promote stemness and chemotherapy resistance of glioma cells, while OPCs’ survival was enhanced by conditioned medium from glioma cells.^[^ ^23^ ^]^ In addition, Huang et al described a subpopulation of OPCs, which were recruited at the tumor border, could promote tumor growth via remodeling vascular network.^[^ ^24^ ^]^ OPCs could also alter the status of microglia homeostasis through downregulation of TGF‐β signaling.^[^ ^25^ ^]^ Interestingly, though glioma cells invading along white matter is a common model of infiltration, this injury‐like microenvironment creates a tumor suppressive feedback loop and slows glioma spread, indicating therapeutic efficacy of myelination‐promoting strategy.^[^ ^26^ ^]^ Therefore, we proposed that OPCs may be a key player in the TME, and tumor cells may encounter opposite signals from senescent and non‐senescent OPCs. What determines the final outcome remains unclear. Preclinical studies reported that RT may promote the accumulation of immunosuppressive cells in TIME by increasing the production of several immunosuppressive cytokines.^[^ ^6^ ^]^ The SASP profile of SnCs, such as IL‐6, IL‐8, CCL2, TNF‐α, and TGF‐β, can be a source of chronic inflammation via the recruitment of immunosuppressive cells. However, the relationship between RT‐induced SASP factors and suppressive TIME formation warrants further study. To address this point, we pre‐irradiated RBO in vitro before co‐culturing them with GBM cells. Interestingly, the tumor‐promoting effects of Sen‐RBO were very limited; this result is contradictory to prior observations in in vivo studies. Then we hypothesized that TIS may promote glioma recurrence via establishment of immunosuppressive TIME rather than directly. Mechanically, our results showed that the production of CXCL12, G‐CSF, TNF‐α, sICAM‐1, and IL‐6 was increased in SnAs compared with normal astrocytes. Furthermore, we observed that TNF‐α derived from SnAs drives CXCL1 production by activating transcriptional activity of c‐Myc‐Max in GBM cells. Increased *CXCL1* expression was closely related to immunosuppressive characteristics in gliomas, possibly via remodeling the TIME composition by recruiting CXCR2+ inflammatory immune cells. However, the exact composition of the SASP profile is mostly dynamic and dependent on cell types, stimulus, and time after treatment. However, the dynamics and biological roles of SnC after anticancer therapy remain largely unknown.

Accumulating evidence supports the presence of heterogeneity among SnCs, warranting high‐specific senolytic drugs for killing these SnCs.^[^ ^12^, ^27^ ^]^ In the present study, we evaluated the levels of antiapoptotic proteins (BCL‐2, BCL‐xL, BCL‐w, and MCL‐1) and prosurvival pathway (PI3K‐AKT signaling pathway) in SnAs. WB results revealed that the protein levels of BCL‐2, BCL‐w, and BCL‐xL and phosphorylated AKT were increased. Therefore, we chose ABT263 and D+Q for subsequent studies. Selective clearance of SnCs via pharmacogenetic (*CDKN2A*‐DTR mice) or pharmacological (ABT263 or D+Q) approaches increased the survival time of tumor‐bearing mice and reshaped the TIME. Interestingly, D+Q mainly induces early apoptosis in SnAs, whereas ABT263 promoted late apoptosis or necrosis at 24 h post treatment. However, the responses of irradiated or mock‐irradiated GBM cells to senolytic drugs varied depending on the cell types.^[^ ^28^ ^]^ More importantly, the efficacy of combined use of RT and senolytic drugs that we reported here supports its therapeutic consideration in the preclinical settings. However, appropriate dosing strategies are still warranted to sustain the efficacy of combination therapy. Moreover, SnCs are quite heterogenous; bystander effects would occur when introducing these agents, and should be aware of high priority. Lastly, the detrimental effects of killing SnCs, including neurons and OPCs, require careful examination in the future.

In conclusion, we propose that IR‐induced SnAs may promote GBM recurrence by remodeling the TIME. Mechanically, TNF‐α derived from SnAs increases CXCL1 production in GBM cells by activating c‐Myc‐Max complex. Clearance of these SnCs by targeting antiapoptotic proteins or prosurvival pathways can enhance the therapeutic efficacy of RT (Figure 8D). Accumulating studies have reported that radiation‐induced changes in the tumor microenvironment may play an important role in GBM recurrence. Leila et al have reported the dynamic changes of TAM populations in tumor microenvironment in response to radiotherapy; blocking IR‐induced alternative activation of TAM by CSF1R inhibition may enhance the initial glioma‐debulking effects.^[^ ^6a^ ^]^ Tracy et al have found that astrocytes could also be affected by IR, and support glioma stemness and survival by transferring Transglutaminase 2.^[^ ^17^ ^]^ In addition, delayed effects of IR on the TME might lead to aggressive tumor regrowth and immunotherapy resistance.^[^ ^29^ ^]^ Though senescent glial cells, including astrocytes, have been discovered in neurodegenerative disease for a long period of time, radiation‐induced astrocyte senescence has recently been identified by co‐immunostaining of P16^INK4A^ and GFAP in irradiated GBM patient tissues.^[^ ^13^ ^]^ Based on these results, Eliot et al have also verified that IR triggered widespread senescence in mouse brains by co‐immunostaining of GFAP and senescence markers. Moreover, the secretion of HGF by senescent astrocytes drives tumor invasion, therapeutic resistance, and recurrence. Proposing that the strategies involving the elimination of senescent cells might achieve higher therapeutic efficacy, they first introduced ABT‐263 for over 25 daily cycles after IR and found that clearance of senescent cells could not only inhibit growth of xenograft glioma cells, but also extend survival of tumor‐bearing mice.^[^ ^11b^ ^]^ GBM cells underwent senescence in response to IR as well. F3 signaling, activated by IR in GBM cells, promotes clonal expansion and global reorganization of immune, ECM, and cytokine landscapes in the TME. Their findings may provide a clue to develop novel therapeutic approach to target senescent tumor cells.^[^ ^30^ ^]^ Interestingly, Rana et al revealed the tumor‐promoting action of oncogene‐induced senescence in GBM. They focused on naturally‐occurring senescence and investigated the pro‐tumorigenic features of resident senescent cells rather than therapy‐induced ones.^[^ ^31^ ^]^ However, IR‐induced senescent cells in the brain and the presence of heterogeneity among those SnCs still lack comprehensive characterization.^[^ ^27a^ ^]^ By introducing snRNA‐seq analysis of irradiated mouse brains, the senescence features of various normal brain cells, which showed distinct expression levels of SASP profiles, were identified and presumably participated in TIME remodeling. In addition, we evaluated the levels of antiapoptotic proteins (BCL‐2, BCL‐xL, BCL‐w, and MCL‐1) and prosurvival pathway (PI3K‐AKT signaling pathway) in SnAs, aiming to discover high‐specific senolytic drugs for killing these SnCs. However, there are still some limitations in our current paper: whole brain irradiation is not used currently for GBM in clinical settings except for the instance of multi‐focal lesions or gliomastosis. As such, a smaller portion of the brain will receive indicated radiation dose. Moreover, the relationship between RT dose response and SnCs induction still remains unclear. In addition, intracranial injection following IR does not consider the alteration of astrocytes by tumor cells, which should also be noticed and investigated in more advanced animal models. Lastly, SASP factors derived from SnAs could also play a crucial role in recruitment of myeloid cells. Annotation of their function in the future could improve our understanding of TIS in tumor progression. Altogether, Our findings provide insights into TIS in the tumor microenvironment, which may have important implications for strategies to combine senolytic drugs with RT and immunotherapy in cancers.

## 4 Experimental Section

### Ethics Statement

All primary and matching recurrent glioma tissue samples (*n* = 12) were obtained from the Department of Neurosurgery at Qilu Hospital of Shandong University. All experiments and the use of human tissues were approved by the Research Ethics Committee of Shandong University and Zhejiang University, and the Ethics Committee of Qilu Hospital of Shandong University and the Second Affiliated Hospital of Zhejiang University School of Medicine (Ethical approval#: 2022‐1076) in accordance with the Declaration of Helsinki (for humans) and the U.S. Public Health Service Policy on Human Care. All animal studies were reviewed and approved by the Institutional Animal Care and Use Committees (IACUC) of the Second Affiliated Hospital of Zhejiang University School of Medicine (Ethical approval#: 2023‐0132), in accordance with the Use of Laboratory Animals (2015 reprint; for mice). Written informed consent was obtained from all adult patients (Table S1, Supporting Information).

### Cell Culture and Reagents

The human GBM cell lines U251 and LN229 were purchased from the American Type Culture Collection (Manassas, VA, USA). The mouse GBM cell lines GL261 and G422 were obtained from iCell Bioscience Inc (Shanghai, China). All cell lines (U251, LN229, GL261, and G422) were maintained in Dulbecco's modified Eagle medium (DMEM) supplemented with 10% fetal bovine serum (Thermo Fisher Scientific; Waltham, MA, USA) and authenticated via short tandem repeat analysis (Cell Cook Biotech Co. Ltd; Guangzhou, China). Normal human astrocytes (HA; ScienCell; Carlsbad, CA, USA) and Mouse astrocytes (MA; ScienCell) were cultured in corresponding Astrocyte Medium BulletKit (ScienCell). Primary GBM#P3 cells and GSC#BG7 were kind gifts provided by Prof. Rolf Bjerkvig (University of Bergen, Norway) and cultured in serum‐free DMEM/F12 medium supplemented with 2% B27 Neuro Mix (Thermo Fisher Scientific), epidermal growth factor (20 ng mL^−1^; Thermo Fisher Scientific), and basic fibroblast growth factor (10 ng mL^−1^; Thermo Fisher Scientific).

### Brain Organoid Cultures and the Invasion Assay

The culture of Rat Brain Organoids (RBO) was performed as previous described.^[^ ^32^ ^]^ After 21 days of culture, normal RBOs were differentiated; then, they were irradiated with a single‐fraction X‐ray dose of 10 Gy to induce senescence.

To establish a coculture invasion ex vivo system of GBM spheroids and RBOs, GBM#P3 and GSC#BG7 cells were seeded into 96‐well plates at 3000 cells/well and incubated for 2 days to form tumor spheroids. At 4 days after irradiation, RBOs were then cocultured with tumor spheroids for 3 days. Finally, images of tumor cell invasion were captured under a confocal microscope (TCS SP8, Leica; Wetzlar, Germany).

### SA‐β‐Gal Staining

To detect SnCs post IR, the SA‐β‐Gal staining kit (Cell Signaling Technology; CST; Beverly, MA, USA) was used according to manufacturer's protocol. Briefly, MA, HA, or RBOs were seeded into six‐well plates and then irradiated at a dose of 10 Gy to induce senescence. At the indicated days, the astrocytes or RBO were fixed with fixative solution and stained with β‐galactosidase staining solution at 37 °C overnight in a dry incubator. Images were captured using a bright‐field microscope (Leica).

### CellTiter‐Glo Assay

To measure the cell viability of GBM cells (GL261, G422, U251, or LN229) or astrocytes (MA or HA), CellTiter‐Glo 2.0 Cell Viability Assay kit (Promega; Madison, WI, USA) was used according to manufacturer's instructions. Further, for testing the pharmacological clearance of SnCs, GBM cells or astrocytes were irradiated at 10 Gy to induce senescence and then treated with either vehicle or senolytic drugs at 4 days after irradiation. After incubating with drugs for 72 h, cells were subjected to CellTiter‐Glo assay.

### Measurement of Apoptosis and Necrosis

To detect apoptosis and necrosis, MA or HA were irradiated at a dose of 10 Gy and incubated with senolytic agents at the indicated concentrations for 24 h and then with RealTime Glo Annexin V Apoptosis Assay (Promega) reagents for another 48 h. Luminescence (annexin V binding) and fluorescence (membrane integrity) intensities were assessed according to the manufacturer's instructions.

### Real‐Time Quantitative RT‐PCR (qRT‐PCR)

Total RNA was extracted from cells using TRIzol Reagent (Thermo Fisher Scientific). qRT‐PCR was performed using a previously described method.^[^ ^33^ ^]^ Briefly, RNA (2 µg) was reverse transcribed into cDNA according to the manufacturer's instructions (Toyobo Life Science; Shanghai, China). Then, qRT‐PCR was performed using the SYBR premix Ex Taq (Takara; Tokyo, Japan) on the Real‐Time PCR Detection System (480II, Roche; Basel, Switzerland). β‐actin was used as an internal control. The primers used for PCR are listed in Table S2 (Supporting Information).

### Single‐Nuclei RNA Sequencing (snRNA‐seq) and Bioinformatic Analysis

For snRNA‐seq, a total of four animals were used: the whole brains from two mice that were irradiated at a dose of 10 Gy (2 Gy × 5 fractions) 28 days previously, were pooled together as one sample. The other two mice that were mock‐irradiated were pooled together as another sample. The nuclei from the frozen brain tissues were isolated using Shbio Nuclei Isolation Kit (SHBIO; Shanghai, China). For each sample, nuclei were counted with a cell counter and immediately loaded onto a Chromium Single Cell Processor (10× Genomics; San Francisco, CA, USA) for RNA barcoding. Finally, two rounds of sequencing were performed using the NovaSeq 6000 sequencing system (Illumina; San Diego, CA, USA).

Using CellRanger Version 5.0.0, the FASTQ files generated from snRNA‐seq were aligned to a custom‐made pre‐mRNA reference that was created according to the instructions of 10× Genomics.

The obtained output was then imported into the Seurat package for quality control and downstream analysis. The normalized data were obtained using the NormalizeData function in the Seurat package for extracting a subset of variable genes. Then, principal component analysis (PCA) was performed, and the data was reduced to the top 30 PCA components after data scaling. Using the Louvain method, graph clustering of the PCA‐reduced data was performed to cluster the cells after preparing a shared nearest‐neighbor graph. For subclustering, the study used the same procedure of data scaling, dimensionality reduction, and clustering to a specific dataset which is usually restricted to one type of cell. For each cluster, the Wilcoxon rank‐sum test was used to identify the significant deferentially expressed genes by comparing the remaining clusters. Further, SingleR and known marker genes were used to identify the cell type. Cell types were identified with a custom‐made list of gene markers (Table S3, Supporting Information). Heatmaps were generated using the scaled data, and violin plots were generated using the normalized data and plotted on a log scale. *CDKN1A‐* and *CDKN2A*‐positive cells were identified based on the presence of at least one *Cdkn1a* or *Cdkn2a* transcript.^[^ ^34^ ^]^ SASP lists were obtained from.^[^ ^14^ ^]^

### Immunohistochemistry (IHC) and Western blotting (WB)

IHC and WB were performed as previously described.^[^ ^35^ ^]^ All antibodies used are described in Supplementary Materials and Methods (Table S4, Supporting Information).

### Immunofluorescence Staining

Immunofluorescence staining of cell cultures was performed using a previously described method.^[^ ^33^ ^]^ To perform immunofluorescence staining of tissue sections, formalin‐fixed, paraffin‐embedded brain sections were deparaffinized and rehydrated. Antigen retrieval was performed in a microwave at 98 °C for 20 min with 1× citrate buffer (pH 6.0) (Sigma‐Aldrich; St Louis, MO, USA). Tissue sections were incubated with the primary antibodyin Superblock (Thermo Fisher Scientific) at 4 °C overnight. Thereafter, the sections were incubated with secondary antibodies for 90 min at room temperature in a light‐resistant, humidified container. Then, the sections were mounted in the dark with DAPI (Invitrogen; Thermo Fisher Scientific). Immunofluorescence images were captured under a confocal microscope (Leica). All the antibodies used are listed in Table S4 (Supporting Information).

### shRNA Treatment

For *CXCL1* knockdown, lentiviral constructs (shRNA; OBiO Technology; Shanghai, China) were used for cell infection. After 48 h, infected cells were cultured in media containing puromycin (2 µg mL^−1^; Thermo Fisher Scientific) for an additional 2 weeks. The shRNA sequences used in this experiment are presented in Table S5 (Supporting Information).

### Fluorescence In Situ Hybridization (RNA‐FISH) and Immune‐RNA‐FISH

The mouse and human probe of *P16^INK4A^* RNA were synthesized by RiboBio Co., Ltd (Guangzhou, China). FISH and immune‐RNA FISH were conducted according to the instructions present in Fluorescent in Situ Hybridization Kit (RiboBio). Briefly, paraffin‐embedded brain sections were first subject to deparaffinization, rehydration, antigen retrieval, and blocking. For fluorescence detection of *P16^INK4A^*, the brain sections were incubated with the probes at 37 °C overnight. Finally, nuclei were counterstained with DAPI (Thermo Fisher Scientific). For immune‐RNA‐FISH, sections were then costained with the antibody against GFAP and secondary antibodies followed by mounting with DAPI. Images were acquired under a confocal microscopy (Leica).

### ELISA

The levels of TNF‐α and CXCL1 proteins were measured using the TNF alpha Mouse ELISA Kit (Invitrogen) and Mouse CXCL1/KC Quantikine ELISA Kit (R&D System; Minneapolis, MN, USA), respectively. Assays were performed according to manufacturers’ instructions.

### Mouse Cytokine Array

To prepare normal MA (Nor‐MA) conditioned medium (CM), MA (3 × 10^5^ cells) were mock‐irradiated and the CM was harvested after 48 h, centrifuged at 1000 x *g* for 5 min to remove cellular debris, and then stored at −80°C. To prepare Sen‐MA‐CM, MA (3 × 10^5^ cells) were irradiated with a single‐fraction X‐ray of 10 Gy to induce cellular senescence. The medium was replaced with fresh medium at 5 days after IR, and the CM was collected after 48 h. To prepare GL261‐CM, GL261 cells were incubated with thawed Control‐/Nor‐MA‐/Sen‐MA‐CM for 48 h, followed by supernatant collection. The collected CMs were subjected to the Proteome Profiler Mouse Cytokine Array Kit (R&D System) according to the manufacturer's instructions. All experiments were performed in triplicate.

### Multiplex Profiling Assay for Transcription Factor Activation

GL261 cells were treated with PBS or recombinant mouse TNF‐α protein for 72 hours. Then, nuclear proteins were isolated and analyzed using 96‐well plate transcription factor activation array (Signosis; Santa Clara, CA, USA) according to the manufacturer's protocol. The activity of each transcriptional factor was normalized to that of the PBS‐treated group.

### Chromatin Immunoprecipitation (ChIP) Assay

The binding sites of c‐Myc on the promotor region of *CXCL1* were predicted using JASPAR (http://jaspar.genereg.net/). Briefly, GL261 cells were plated into 10‐cm dishes and treated with PBS or recombinant mouse TNF‐α protein for 48 h. ChIP assay was performed using the SimpleChIP Plus Enzymatic Chromatin IP Kit (Magnetic Beads, #9005, CST) and anti‐c‐Myc (#18583, CST) according to the manufacturer's instructions. The final ChIP DNA samples were then used as templates for qPCR. The primers used for PCR are listed in Table S2 (Supporting Information).

### Dual Luciferase Reporter Assays

The *firefly* and *renilla* luciferase reporter (100 ng each) were cotransfected using Lipofectamine 3000 (Thermo Fisher Scientific). Luciferase activity was measured after 48 h using the Dual‐Luciferase Reporter Assay System (Promega). *Firefly* luciferase activity was normalized by *renilla* luciferase activity in the same well. Assays were performed on cells in three wells for each experiment to obtain an average count, and in three independent biological replicates.

The Myc‐Max responsive luciferase reporter plasmid (Myc‐Luc reporter), and CXCL1 promoter luciferase reporter plasmids (pGL3‐CXCL1‐Wild‐type [WT]/DEL1/DEL2) were synthesized by Genomeditech Co., Ltd (Shanghai, China).

### Animal Studies

WT C57BL/6J (female, aged 4–6 weeks) were purchased from SiPeiFu Biotechnology Co., Ltd (Beijing, China). *CDKN2A*‐DTR mice were generated by crossing *Rosa26‐LSL‐iDTR* (Strain#: 007900, Jackson Lab, USA) with *CDKN2A‐Luc‐tdTomato‐Cre^ERT2^* (Shanghai Model Organisms Center, Inc, Shanghai, China). Athymic nude mice (female, aged 4–6 weeks) were purchased from GemPharmatech Co., Ltd (Nanjing, China). Mice were restrained on a platform attached to a treatment couch and cranially irradiated in the anterior‐posterior position using an X‐ray device (X‐RAD225 OptiMAX, Precision X‐ray; 225 kV, 13.3 mA, 2.05 Gy min^−1^, and 2‐mm Al filter) fitted with a specifically designed collimator that provided a 20 mm‐diameter field size for iso‐dose exposure. The body of mice outside of the irradiation field was protected using a shielding device. The mice in control group were mock‐irradiated. Mice were periodically monitored for weight loss and other symptoms of mucositis. All mice were provided with extra gel food packs.

For intracranial stereotactic injections, GL261 (1 × 10^4^ per mouse) or G422 (2 × 10^2^ per mouse) cells were suspended in PBS (5 µl) and delivered into frontal lobes of mice using a stereotactic apparatus (KDS310, KD Scientific; Holliston, MA, USA) as described previously.^[^ ^23^ ^]^

For pharmacological studies, 14 days post cranial irradiation, mice were treated with ABT263 (50 mg kg^−1^, MCE; NJ, USA) or dasatinib (5 mg kg^−1^, MCE) plus quercetin (50 mg k^−1^g, MCE) or vehicle (60% Phosal, 10% ethanol, and 30% PEG‐400) by oral gavage every other day for a total of seven doses, followed by IC injection of GBM cells.

For pharmacogenetic studies, tamoxifen (TMX; 100 mg kg^−1^, Sigma‐Aldrich) dissolved in corn oil (Sigma‐Aldrich) at a concentration of 20 mg ml^−1^ was intraperitoneally injected into *CDKN2A*‐DTR mice at 14 days post cranial irradiation for 5 consecutive days, followed by administration of diphtheria toxin (DT; 15 g/kg, Sigma‐Aldrich) every other day for a total of three doses via intraperitoneal injection. Mice were subjected to in vivo bioluminescence imaging (Perkin Elmer, Hopkinton, MA, USA) every week.

For therapeutic efficacy studies, GL261 (1 × 10^5^ per mouse), G422 (1 × 10^5^ per mouse), LN229 (3 × 10^5^ per mouse), or U251 (3 × 10^5^ per mouse) cells were suspended in PBS (5 µl) and delivered into frontal lobes of mice. 7 days after implantation, mice were mock‐irradiated or irradiated with five IRs of 2 Gy each. Then, 28 days after implantation, senolytic agents such as ABT263 or D+Q were orally administrated to mice every other day for a total of seven doses.

Tumor‐bearing mice were sacrificed at the end of the experimental period or when they displayed symptoms, such as apathy, decreased activity, severe hunchback posture, dragging legs, unkempt fur, or body weight loss. Mice were perfused with 0.9% NaCl and then with 4% paraformaldehyde. The brains were excised and prepared for further examination via immunofluorescence staining. All animal studies were reviewed and approved by the Institutional Animal Care and Use Committees (IACUC) of Zhejiang University.

### Statistical Analysis

All experiments were performed using at least three independent biological replicates and reported as mean ± standard error of mean. Unpaired two‐tailed Student's *t*‐test for direct comparisons and ANOVA for multigroup comparisons were performed using GraphPad Prism version 8.00 software. Survival curves were estimated using the Kaplan–Meier method and compared using the log‐rank test. A *P*‐value < 0.05 was considered statistically significant.

### Data Availability

The snRNA‐seq data generated and analyzed in the current study will be available in the NCBI GEO repository after publication, under the accession codes GSE222670.

## Conflict of Interest

The authors declare no conflict of interest.

## Author Contributions

J.J. and K.D. contributed equally to this work and shared co‐first authorship. J.J. and K.D. conceived and designed the project. J.J., K.D., B.C., X.Z., and T.L. performed experiments. J.J., K.D., and B.C. analyzed the data. J.J. and K.D. wrote the manuscript. B.H., H.Y., Y.C., X.X., H.L., T.W., Jiayin Z., D.Y., F.L., C.W., J.C., F.Y., L.W., and Jianmin Z. provided reagents and materials. M.J., A.L., and Jianmin Z. gave intellectual input. J.J. and K.D. revised the manuscript. G.C., X.L., J.W., and S.Y. supervised the study.

## Supporting information

Supporting Information

Supporting Information

## Data Availability

The data that support the findings of this study are openly available in Repository name https://www.ncbi.nlm.nih.gov/geo/query/acc.cgi?acc=GSE222670 Reference number provided by the repository.
