# Supplementary material for: Radiotherapy‐Induced Astrocyte Senescence Promotes an Immunosuppressive Microenvironment in Glioblastoma to Facilitate Tumor Regrowth
Source: Adv Sci (Weinh). 2024 Feb 11;11(15):2304609. doi: 10.1002/advs.202304609 (PMC11022718; doi:10.1002/advs.202304609)
Supplement: Supplementary file 1 — Supporting Information [file ADVS-11-2304609-s002.pdf]

ADVANCED SCIENCE
Open Access

Supporting Information

for *Adv. Sci.*, DOI 10.1002/advs.202304609

Radiotherapy-Induced Astrocyte Senescence Promotes an Immunosuppressive Microenvironment in Glioblastoma to Facilitate Tumor Regrowth

Jianxiong Ji, Kaikai Ding, Bo Cheng, Xin Zhang, Tao Luo, Bin Huang, Hao Yu, Yike Chen, Xiaohui Xu, Haopu Lin, Jiayin Zhou, Tingtin Wang, Mengmeng Jin, Aixia Liu, Danfang Yan, Fuyi Liu, Chun Wang, Jingsen Chen, Feng Yan, Lin Wang, Jianmin Zhang, Senxiang Yan*, Jian Wang*, Xingang Li* and Gao Chen**

Supplementary information for

Radiotherapy-induced astrocyte senescence promotes an immunosuppressive microenvironment in glioblastoma to facilitate tumor regrowth

Supplementary Figure S1

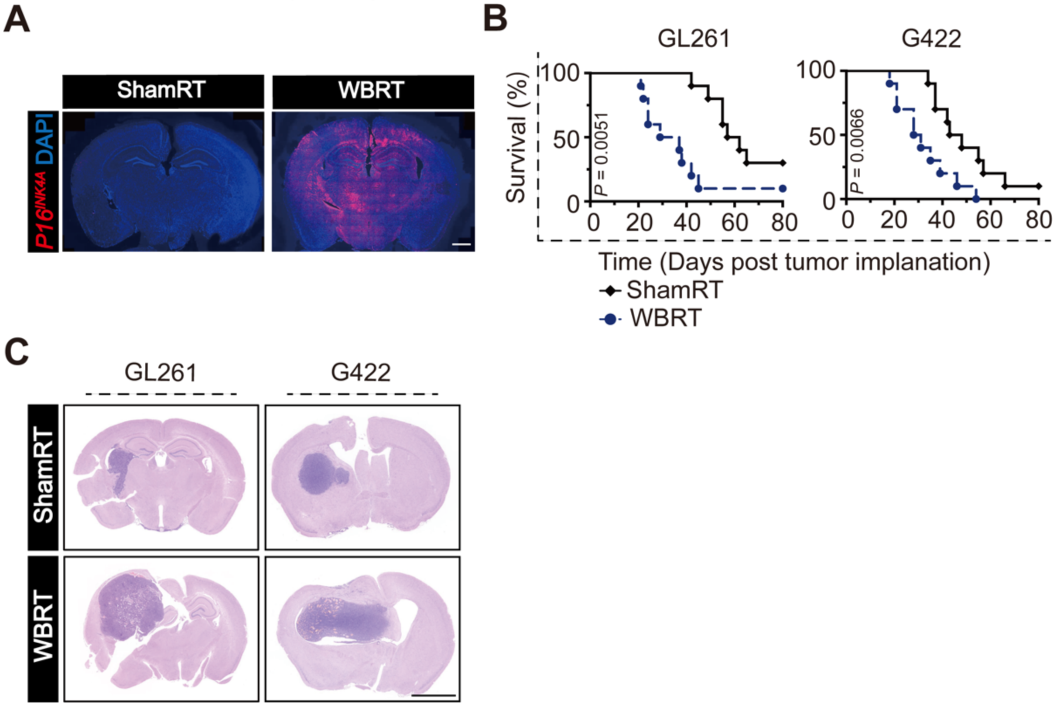

Supplementary Fig. S1. (A) Representative images of *P16*^{INK4A} RNA FISH of indicated mouse brains. Scale bar, 1000 µm. (B) Kaplan-Meier graphs showing the survival time of irradiated or mock-irradiated mice with orthotopic implantation of GL261 and G422 xenografts at indicated timepoints after IR (n = 10 per group); log-rank test. (C) Representative images of H&E staining of GL261- and G422-derived xenografts. Scale bar, 2000 µm.

Supplementary Figure S2

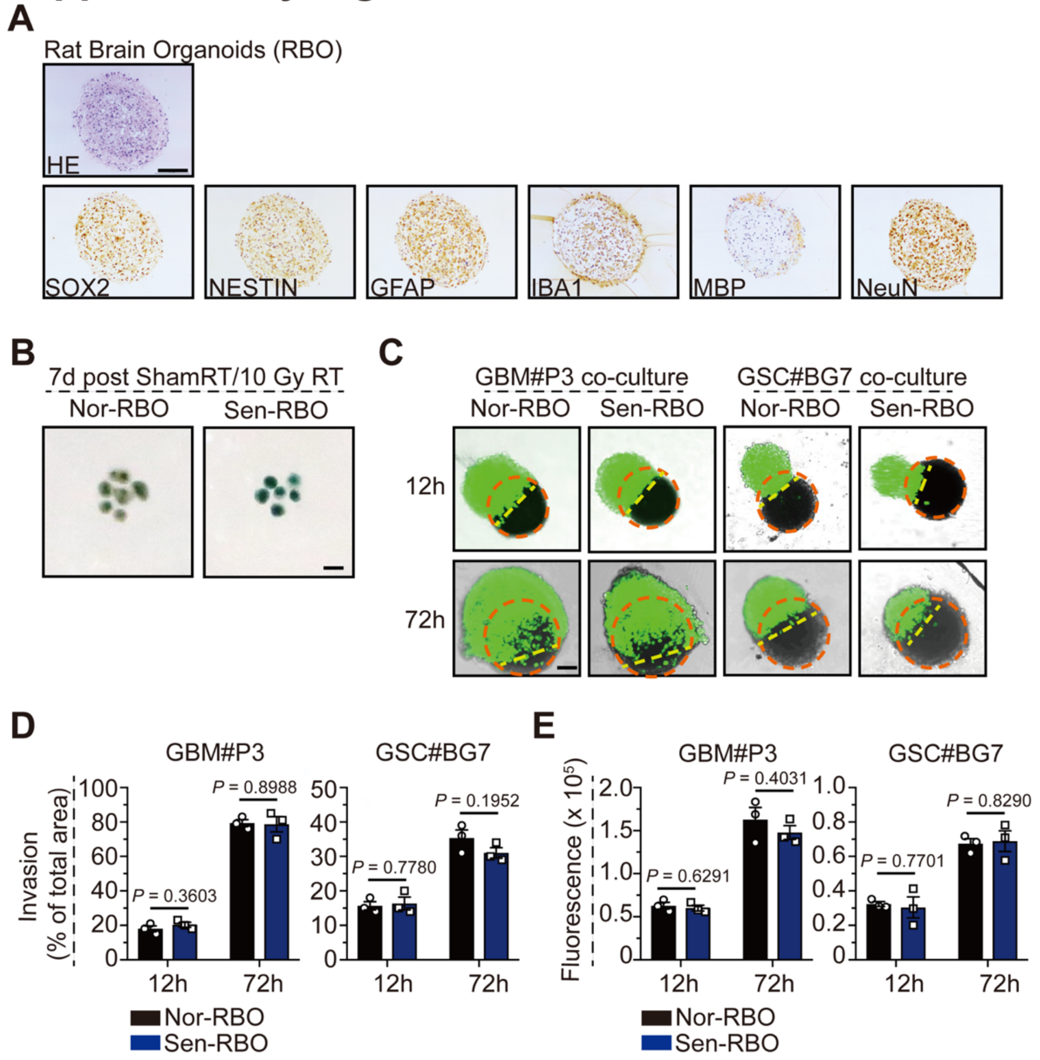

Supplementary Fig. S2. (A) Characteristics of rat brain organoids (RBOs) used in this study. Scale bar, 1000 µm. (B) Representative images of senescence-associated β-galactosidase staining of irradiated or mock-irradiated RBOs at 7 days post IR. Scale bar, 2000 µm. (C, D) Representative images (C) and quantification (D) of RBO-GBM invasion assay evaluated at 12 and 72h after coculture. GBM cells are GFP-tagged. Scale bar, 100 µm. (E) Immunofluorescence intensity was evaluated by microplate reader.

Supplementary Figure S3

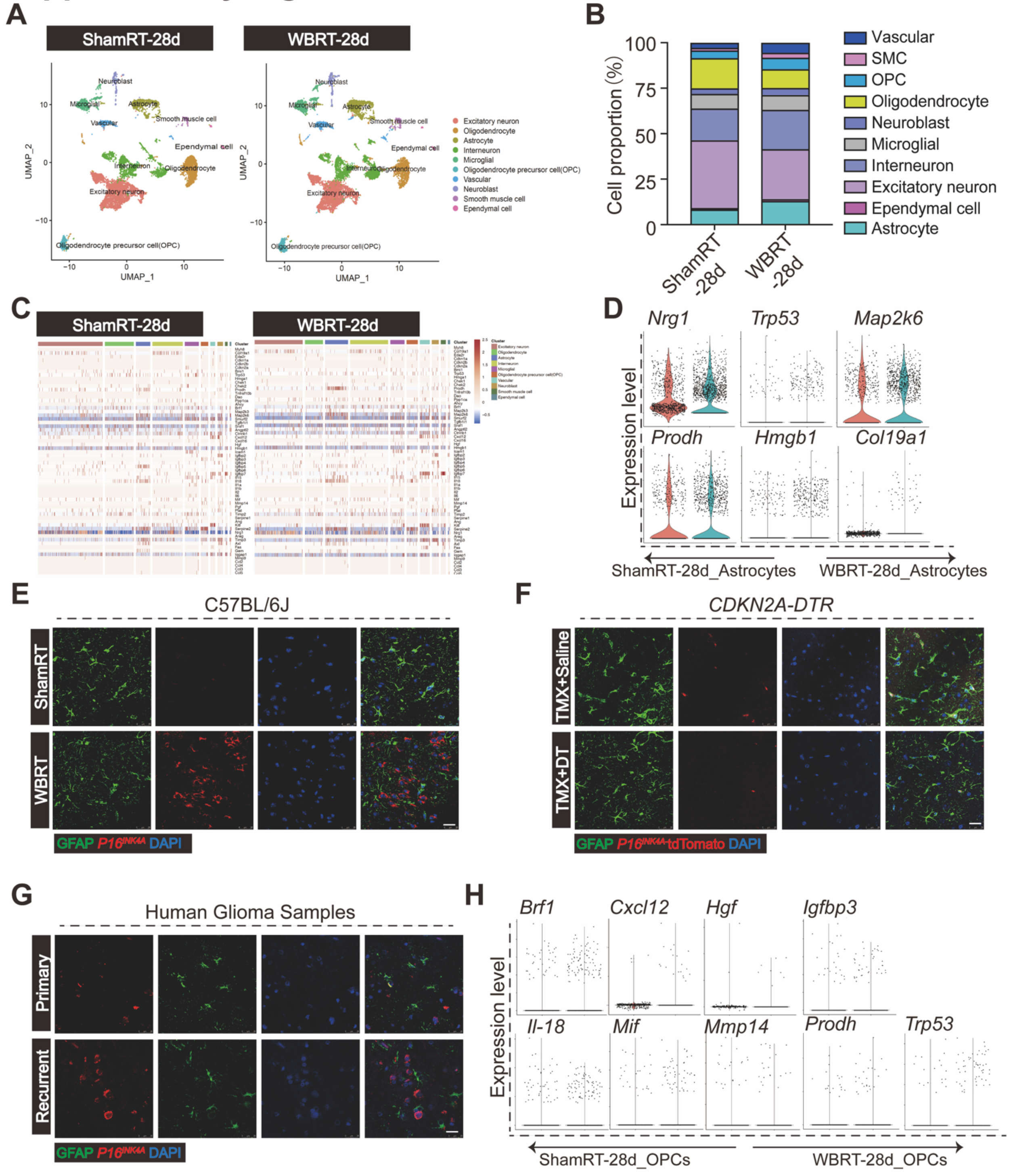

Supplementary Fig. S3. (A) UMAP embeddings of snRNA-seq. Colors represent cell type annotations. (B) Bar graph showing fractions of cell populations isolated from irradiated or mock-irradiated mice. (C) Heatmaps showing expression of SASP factors in various types of brain cells identified in above snRNA-seq dataset. (D) Violin plots showing expression of SASP factors in astrocytic population from irradiated (Right side in the violin plots; blue) or mock-irradiated (Left

side in the violin plots; red) mice. (*Nrg1*: *FDR* = 2.91E-06; *Trp53*: *FDR* = 3.92E-02; *Col19a1*: *FDR* = 1.00E-01; *Map2k6*: *FDR* = 1.28E-01; *Prodh*: *FDR* = 1.83E-01; *Hmgb1*: *FDR* = 2.66E-01; *Timp2*: *FDR* = 3.47E-01). (E) Representative images of immune-RNA FISH against $P16^{INK4A}$ and GFAP in the brain sections of wild-type C57BL6/J mice. Scale bar, 25 µm. (F) Representative images of $P16^{INK4A}$-tdTomato-positive astrocytes (GFAP+) in the brain sections from *CDKN2A*-DTR mice. Scale bar, 25 µm. (G) Representative images of immune-RNA FISH against $P16^{INK4A}$ and GFAP in paired primary and recurrent glioma samples (n = 12). Scale bar, 25 µm. (H) Violin plots showing expression of SASP factors in OPCs population from irradiated (Right side in the violin plots; blue) or mock-irradiated (Left side in the violin plots; red) mice. (*Mmp14: FDR* = 0.178498519; *Cxcl12: FDR* = 0.390146289; *Trp53: FDR* = 0.929615571; *Brf1: FDR* = 1; *Hgf: FDR* = 1; *Igfbp3: FDR* = 1; *Prodh: FDR* = 1; *Il-18: FDR* = 1; *Mif: FDR* = 1).

Supplementary Figure S4

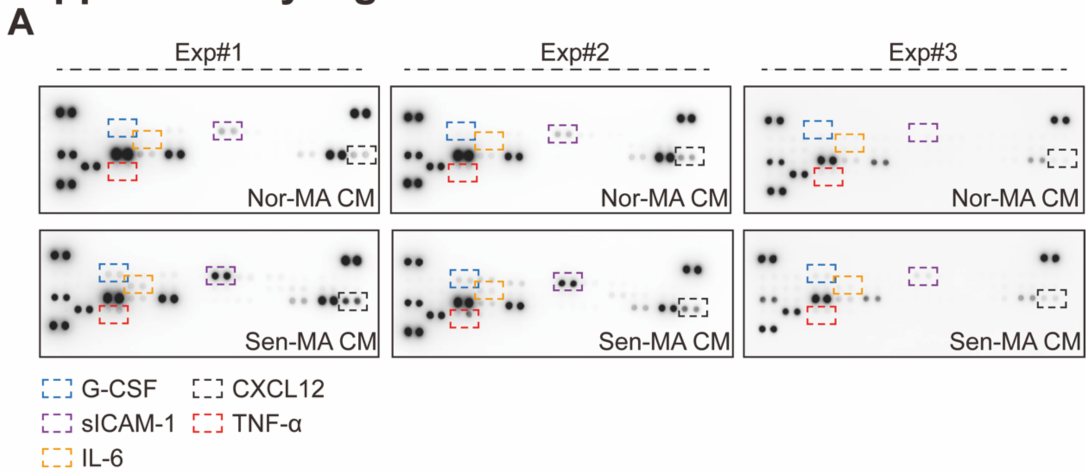

Supplementary Fig. S4. (A) Images of multiple cytokine array analysis of conditioned medium from irradiated and mock-irradiated MA. Colors represent indicated cytokines.

Supplementary Figure S5

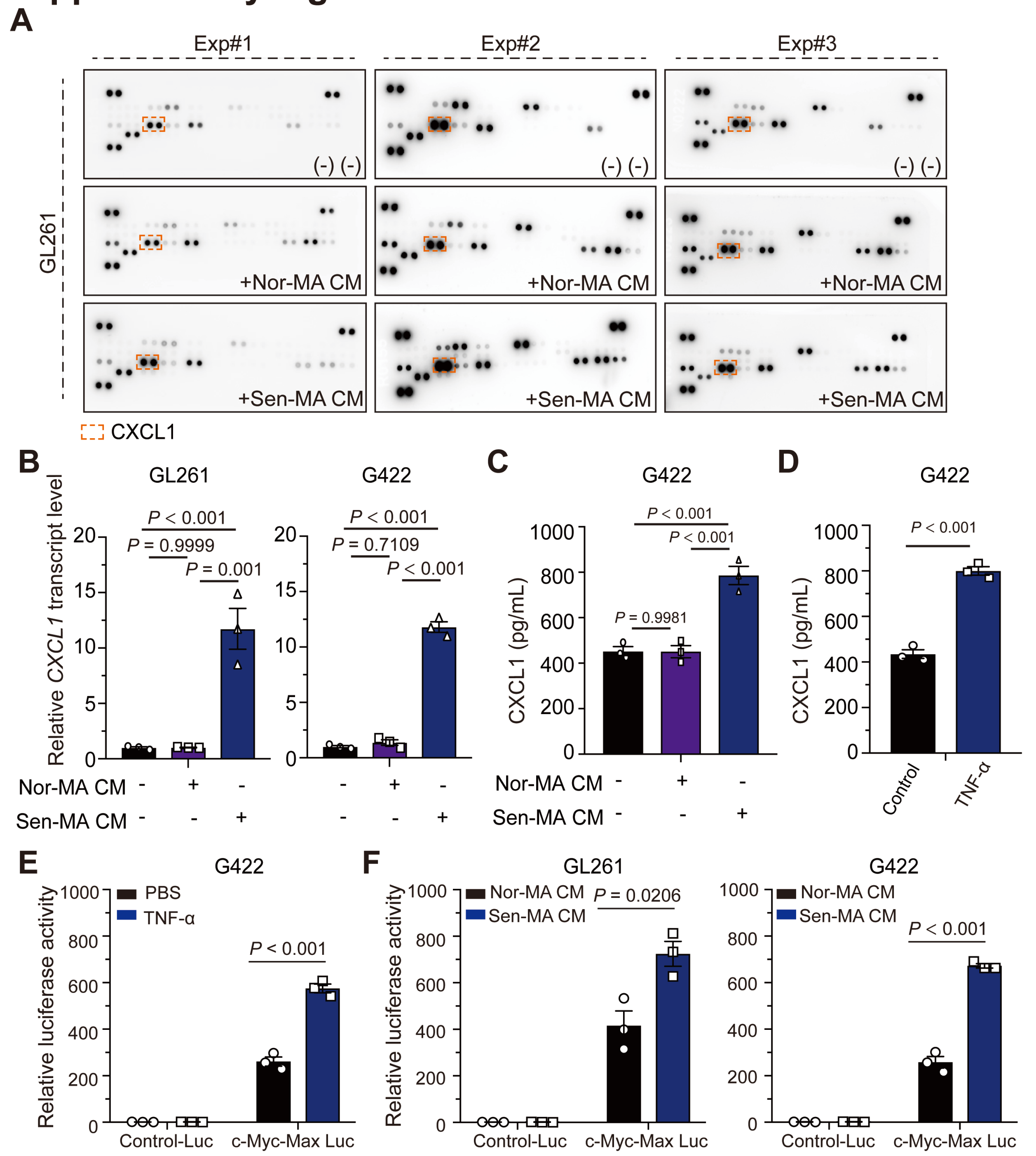

Supplementary Fig. S5. (A) Images of multiple cytokine array analysis of indicated conditioned medium from GL261 cells. Colors represent indicated cytokines. (B) *mRNA* was extracted and subjected to qRT-PCR. *β-actin* was used as internal control. (C) CXCL1-ELISA revealed the stimulation of CXCL1 production in G422 cells by incubation with Sen-MA-CM. (D) CXCL1 production in G422 cells was increased following TNF-α treatment at the concentration detected in Sen-MA-CM (80 pg/mL). (E) G422 cells were transfected with control or c-Myc-Max promoter

firefly luciferase constructs along with renilla luciferase reporters, followed by TNF-α treatment for 48 h. Firefly luciferase activity was normalized by renilla luciferase activity and compared with the control group. (F) GL261 and G422 cells were transfected with control or c-Myc-Max promoter *firefly* luciferase constructs along with *renilla* luciferase reporters, followed by Nor-MA or Sen-MA CM for 48 h. *Firefly* luciferase activity was normalized by *renilla* luciferase activity and compared with the control group.

Supplementary Figure S6

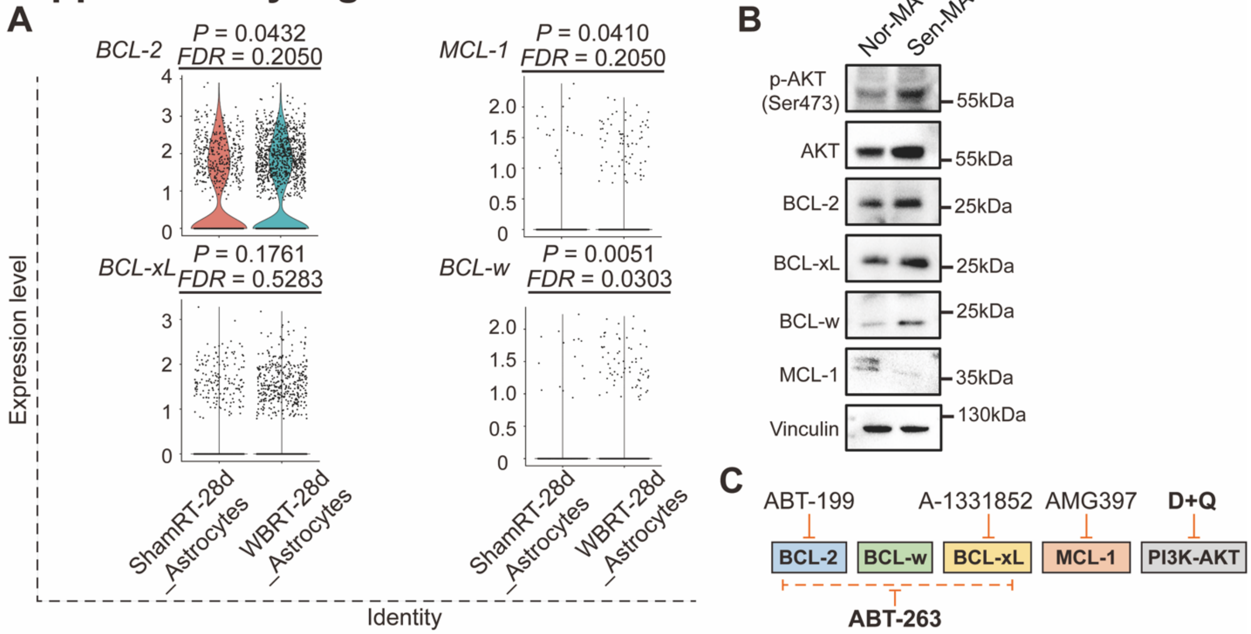

Supplementary Fig. S6. (A) Violin plots showing expression of *BCL-2*, *BCL-w*, *MCL-1* and *BCL-xL* in astrocytic population from irradiated or mock-irradiated mice. (B) Western blot analysis of p-AKT (Ser473), AKT, BCL-2, BCL-xL, BCL-w, MCL-1 and Vinculin proteins in lysates prepared from GL261 cells. (C) A schematic model of senolytic drugs and their potential targets accordingly.

Supplementary Figure S7

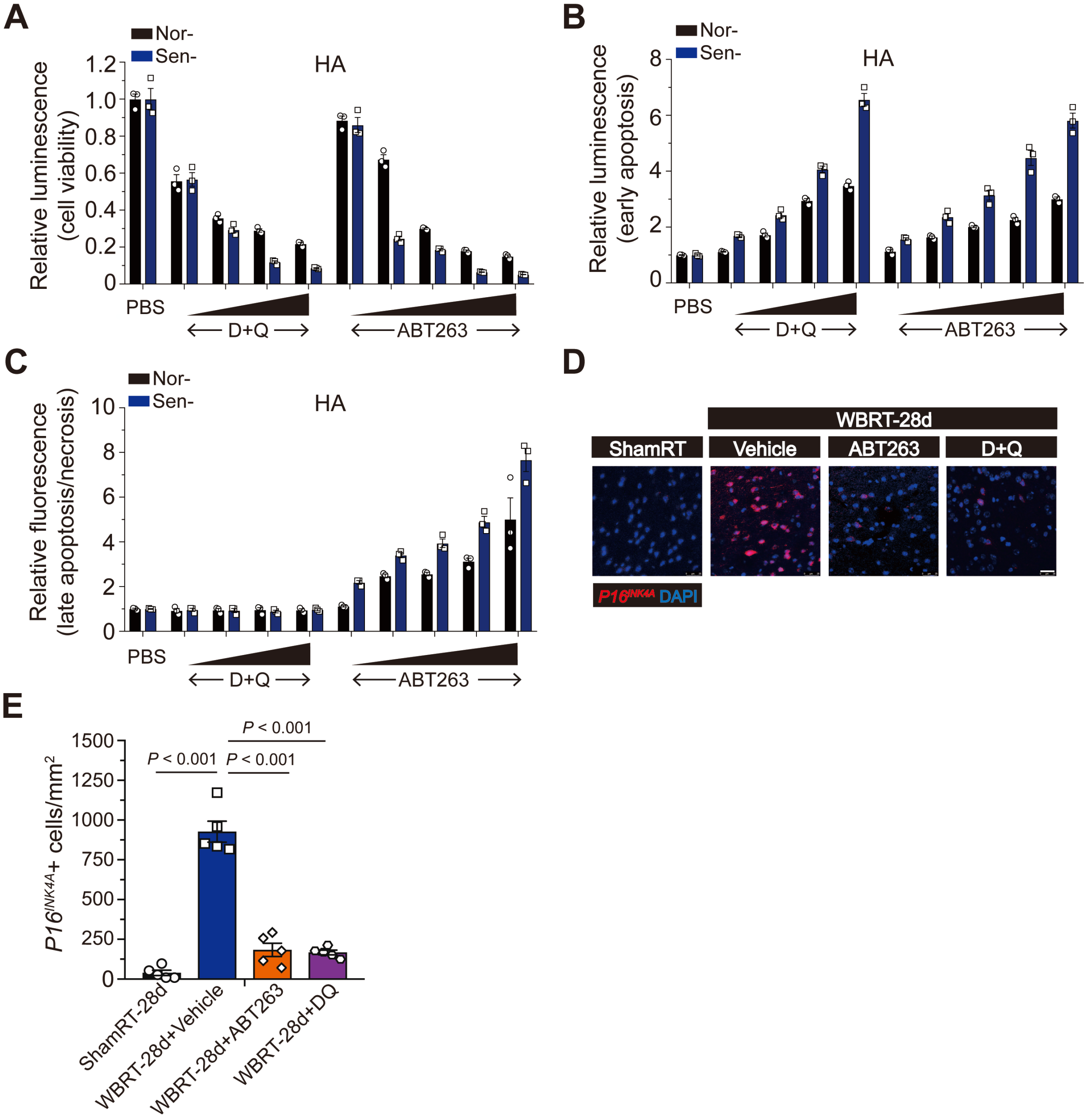

Supplementary Fig. S7. (A) Nor-/Sen-HA were incubated with gradient concentrations of ABT263 (1, 5, 20, 50, 100 μM) or D+Q (1/20, 10/20, 10/40, 20/40 μM) for 72 h and subjected to CellTiter Glo assay. (B, C) Nor-/Sen-HA were incubated with gradient concentrations of ABT263 (1, 5, 20, 50, 100 μM) or D+Q (1/20, 10/20, 10/40, 20/40 μM) for 24 h and subjected to RealTime-Glo Annexin V Apoptosis Assay. (D, E) Representative images (D) and quantification (E) of *P16*^{INK4A} RNA FISH of mouse brains at indicated time points. Scale bar, 25 μm.

Supplementary Figure S8

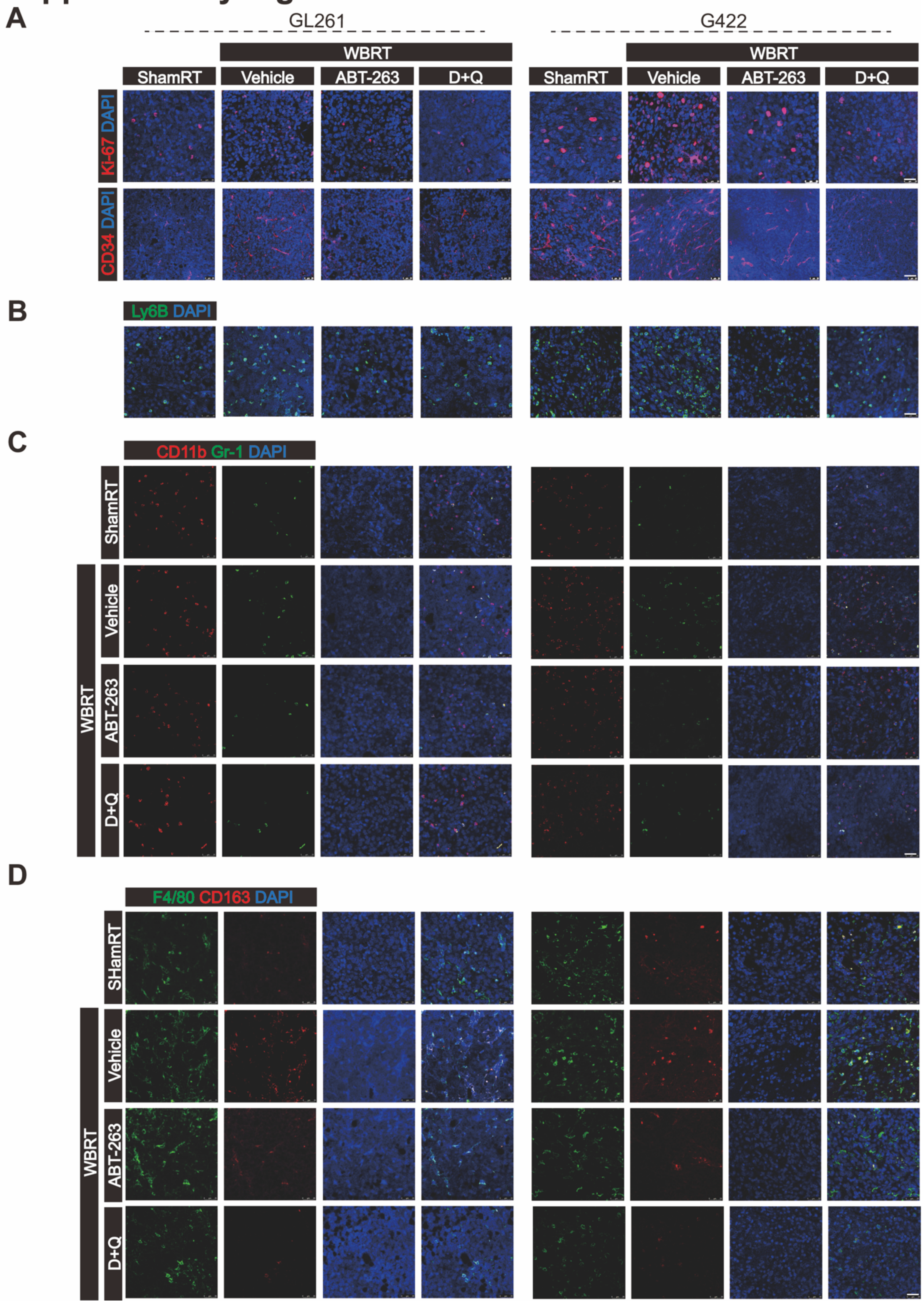

Supplementary Fig. S8. (A, B, C, D) Representative images of (A) Ki-67, CD34, (B) Ly6B, (C) CD11b and Gr-1, (D) F4/80 and CD163, immunofluorescence staining of GL261- and G422-derived xenografts. Scale bar, 25 μm.

Supplementary Figure S9

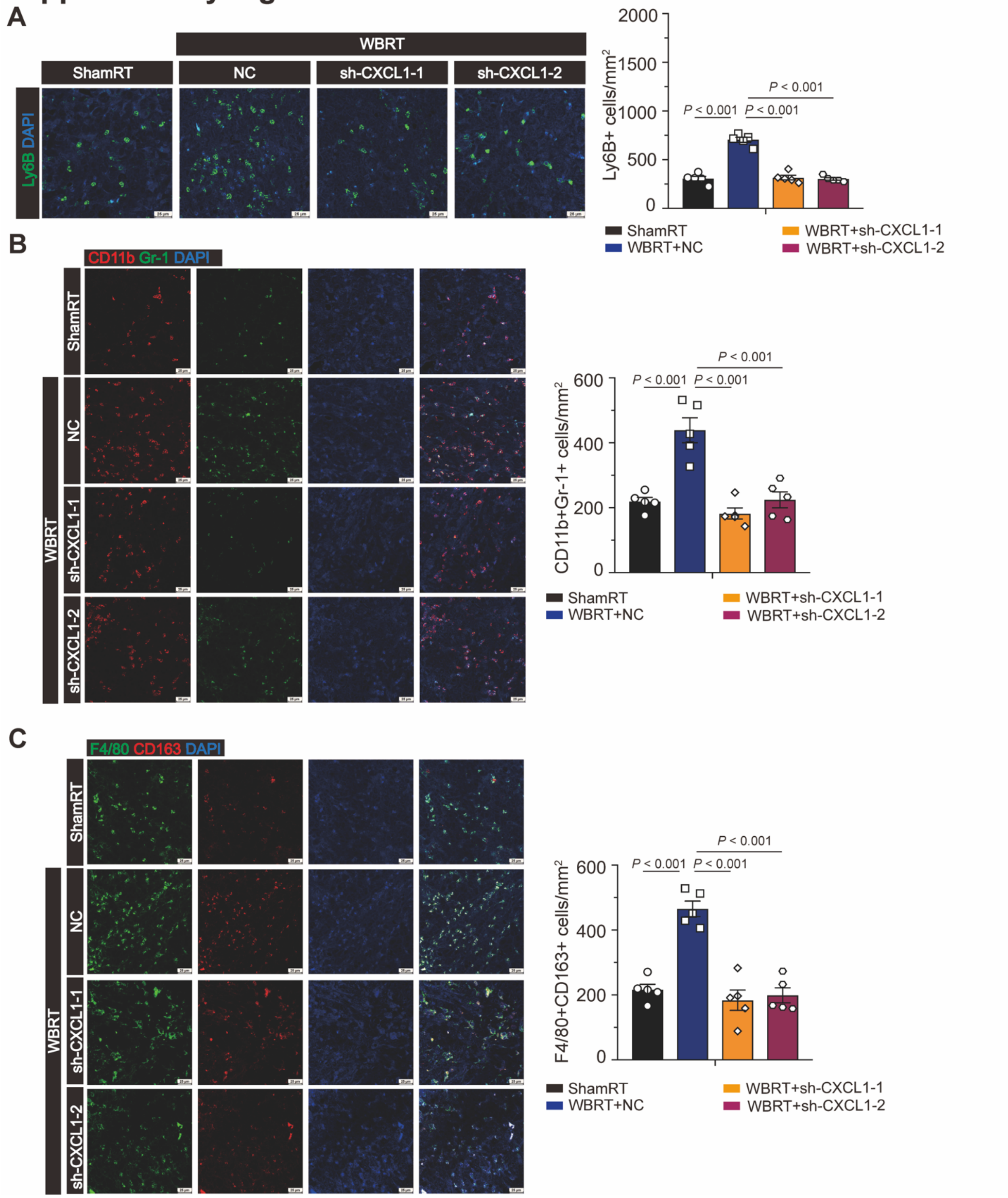

Supplementary Fig. S9. (A, B, C) Representative images and quantification of (A) Ly6B, (B) CD11b and Gr-1, (C) F4/80 and CD163, immunofluorescence staining of GL261-derived xenografts. Scale bar, 25 μm.
