# Supplementary material for: Radiotherapy‐Induced Astrocyte Senescence Promotes an Immunosuppressive Microenvironment in Glioblastoma to Facilitate Tumor Regrowth
Source: Adv Sci (Weinh). 2024 Feb 11;11(15):2304609. doi: 10.1002/advs.202304609 (PMC11022718; doi:10.1002/advs.202304609)
Supplement: Supplementary file 2 — Supporting Information [file ADVS-11-2304609-s001.pdf]

ADVANCED SCIENCE
Open Access

Supporting Information

for *Adv. Sci.*, DOI 10.1002/advs.202304609

Radiotherapy-Induced Astrocyte Senescence Promotes an Immunosuppressive Microenvironment in Glioblastoma to Facilitate Tumor Regrowth

Jianxiong Ji, Kaikai Ding, Bo Cheng, Xin Zhang, Tao Luo, Bin Huang, Hao Yu, Yike Chen, Xiaohui Xu, Haopu Lin, Jiayin Zhou, Tingtin Wang, Mengmeng Jin, Aixia Liu, Danfang Yan, Fuyi Liu, Chun Wang, Jingsen Chen, Feng Yan, Lin Wang, Jianmin Zhang, Senxiang Yan*, Jian Wang*, Xingang Li* and Gao Chen**

Supplementary Table S1 Patient information

Case#	Sex	Age	Initial treatment	PFS	Recurrent treatment	Pathology information
Patient 1	Male	50 yo	Surgery; Radiation	7m	Surgery	1. Right frontal and parietal lobes; GBM; WHO IV;<br>2. Right frontal lobe; Gliosarcoma; WHO IV; GFAP (+), Vimentin (+), P53 (+), Ki-67% > 90%.
Patient 2	Male	46 yo	Surgery; Radiation	6m	Surgery; Apatinib	1. Left frontal lobe; GBM; GFAP (+), Vimentin (+), SMA (+), Ki-67% 15-60%;<br>2. Frontal lobe; GBM; GFAP (+), EGFR (+), P53 (+), SMA (-), Ki-67% > 60%.
Patient 3	Male	48 yo	Surgery; Radiation	122m	Surgery; Radiation; TMZ	1. Left frontal lobe; Anaplastic Oligodendroglioma;<br>2.Left frontal lobe; Anaplastic astrocytoma; WHO II; GFAP (+), Olig2 (+), IDH-1 (+), S-100 (+), P53 (-), EGFR (+), Ki67% = 3%.
Patient 4	Female	43 yo	Surgery; Radiation	12m	Surgery	1. Right temporal lobe; GBM; GFAP (+), P53 (+), EGFR (+), Ki-67% = 20%.<br>2. Right temporal and parietal lobes; GBM; WHO IV; GFAP (+), Ki-67% = 3/15%.
Patient 5	Male	47 yo	Surgery; Radiation	9m	Surgery; Radiation; TMZ	1. Right temporal lobe; Astrocytoma; WHO II;<br>2. Right temporal lobe; GBM; WHO IV; GFAP (+), P53 (-), Ki-67% = 20~30%.
Patient 6	Male	58 yo	Surgery; Radiation	60m	Surgery; Radiation	1. Left parietal lobe; Anaplastic astrocytoma; WHO III; NOS.<br>2. Left parietal lobe; Anaplastic astrocytoma; WHO III; NOS.

Supplementary Table S2 PCR and ChIP-PCR primers used in this study

Primer set	Primers	Sequence (5'-3')	Product size (bp)
CXCL12	F<br>R	5'-TTCTTCGAGAGCCACATCGC-3'<br>5'-GGGCACAGTTTGGAGTGTTG-3'	70
G-CSF	F<br>R	5'-CAGGGTTTCCTGTCCTCACA-3'<br>5'-TCAATTCCGGGGCTAAACCT-3'	72
TNF-α	F<br>R	5'-AGCCCATGGGTTGTACCTTG-3'<br>5'-ATAGCAAATCGGCTGACGGT-3'	99
sICAM-1	F<br>R	5'-CAAAGCTCGACACCCCTGAC-3'<br>5'-GTTTGTGCTCTCCTGGGTCG-3'	140
IL-6	F<br>R	5'-CTTCTTGGGACTGATGCTGGT-3'<br>5'-CTCTGTGAAGTCTCCTCTCCG-3'	73
CXCL1	F<br>R	5'-GGCTGGGATTCACCTCAAGAA-3'<br>5'-TGAGTGTGGCTATGACTTCGG-3'	89
Myc	F<br>R	5'-GTCTTTCCCTACCCGCTCAA-3'<br>5'-TTCTTGCTCTTCTTCAGAGTCGC-3'	189
β-actin	F<br>R	5'-GGCTGTATTCCCCTCCATCG-3'<br>5'-CCAGTTGGTAACAATGCCATGT-3'	154
CXCL1 (site -978/971, ChIP)	F<br>R	5'-TATCTAAGTCTGGTGTATTGGCTC-3'<br>5'-CTATTGAACTTCCAAGCCTTCTCCT-3'	78

Supplementary Table S3 Cell type annotation and gene markers used in snRNA-seq

Cell type annotation	Gene markers
Excitatory neuron	*Camk2a, Grin1, Grin2a, Rbfox3, Syt1*
Oligodendrocyte	*Mbp, Mog, Mobp, Plp1*
Astrocyte	*Pax6, Tnc, Slc1a3, Aqp4, Arhgef26, Bmpr1b*
Interneuron	*Gad1, Gad2*
Microglia	*Ptprc, Ctss, Abi3, Adgre1, CD84*

OPC	*Gspg4, Gpr17, Cdo1*
Vascular cell	*Flt1, Pecam1, Tek*
Neuroblast	*Eya2, Dcn, lgf2, Col3a1*
Smooth muscle cell	*Atp13a5, Abcc9, Slc38a11*
Ependymal cell	*1700007G11Rik, Ak7, Ak9, Arma3, Ccdc108, Daw1, Dnah11*

Supplementary Table S4 Table of antibodies used in this study

Antibody	Catalog Number	Vendor	Dilution
Ki-67	#ab15580	Abcam	IF 1:400
CD34	#ab81289	Abcam	IF 1:200
Ly6B	#MA5-16541	Invitrogen	IF 1:100
CD11b	#ab184308	Abcam	IF 1:100
Gr-1	# RB6-8C5	eBioscience	IF 1:100
F4/80	#ab6640	Abcam	IF 1:100
CD163	#ab182422	Abcam	IF 1:100
γ-H2AX	#05-636	Merck	IF 1:400 (Mus)
	#9718	CST	IF 1:200 (Homo)
SOX2	#ab92494	Abcam	IHC 1:200
Nestin	#ab6142	Abcam	IHC 1:200
GFAP	#16825-1-AP	Proteintech	IHC 1:200<br>IF 1:200
IBA1	#ab178846	Abcam	IHC 1:200
MBP	#78896	CST	IHC 1:200
NeuN	#ab177487	CST	IHC 1:200
p-AKT (Ser 473)	#66444-1-AP	Proteintech	WB 1:1000
AKT	#4691	CST	WB 1:1000
BCL-2	#3498	CST	WB 1:1000
BCL-xL	#2764	CST	WB 1:1000

BCL-w	#16026-1-AP	Proteintech	WB 1:1000
MCL-1	#5453	CST	WB 1:1000
Vinculin	# ab129002	Abcam	WB 1:10000
c-Myc	#18583	CST	CHIP 1:100<br>WB 1:1000
Max	#4739	CST	WB 1:500
TNF-α	#16-7321-81	eBioscience	Neu
TNFR1	#16-1202-81	eBioscience	Neu

Supplementary Table S5 Oligonucleotide sets used in this study

shRNAs	Sequences
sh-NC	5'-TTCTCCGAACGTGTCACGT-3'
sh-*CXCL1*-1	5'-GCAGACCATGGCTGGGATTCA-3’
sh-*CXCL1*-2	5'-GCACCCAAACCGAAGTCATAG-3’

Supplementary Table S6 Reagents for *in vitro* and *in vivo* studies

Reagent	Catalog Number	Vendor
ABT-263	#HY-10087	MCE
Dasatinib<br>quercetin	#HY-10181<br>#HY-18085	MCE
CXCL12	# HY-P7285	MCE
G-CSF	#250-05	PEPROTECH
TNF-α	#HY-P7090	MCE
sICAM-1	#553004	Biolegend
IL-6	#HY-P7063	MCE
MYCi975	# T12133	TOPSCIENCE
